# Theoretical Investigation by DFT and Molecular Docking of Synthesized Oxidovanadium(IV)-Based Imidazole Drug Complexes as Promising Anticancer Agents

**DOI:** 10.3390/molecules27092796

**Published:** 2022-04-27

**Authors:** Amal S. Basaleh, Fatimah Y. Alomari, Abeer A. Sharfalddin, Najlaa S. Al-Radadi, Doaa Domyati, Mostafa A. Hussien

**Affiliations:** 1 Department of Chemistry, Faculty of Science, King Abdulaziz University, P.O. Box 80203, Jeddah 21589, Saudi Arabia; abasaleh@kau.edu.sa (A.S.B.); sharfalddin.aa@hotmail.com (A.A.S.); 2 Chemistry Department, College of Science, Imam Abdulrahman Bin Faisal University, P.O. Box 76971, Dammam 31441, Saudi Arabia; falumary@stu.kau.edu.sa; 3 Department of Chemistry, Faculty of Science, Taibah University, P.O. Box 30002, Al-Madinah Al-Munawarah 14177, Saudi Arabia; nsa@taibahu.edu.sa; 4 Department of Chemistry, College of Science, University of Jeddah, P.O. Box 80327, Jeddah 21589, Saudi Arabia; dmdomyati@uj.edu.sa; 5 Department of Chemistry, Faculty of Science, Port Said University, Port Said 42521, Egypt

**Keywords:** oxidovanadium(IV) complexes, imidazole drugs, DFT study, DNA-binding, molecular docking, anticancer activity

## Abstract

Vanadium compounds have been set in various fields as anticancer, anti-diabetic, anti-parasitic, anti-viral, and anti-bacterial agents. This study reports the synthesis and structural characterization of oxidovanadium(IV)-based imidazole drug complexes by the elemental analyzer, molar conductance, magnetic moment, spectroscopic techniques, as well as thermal analysis. The obtained geometries were studied theoretically using density functional theory (DFT) under the B3LYP level. The DNA-binding nature of the ligands and their synthesized complexes has been studied by the electronic absorption titrations method. The biological studies were carried with in-vivo assays and the molecular docking method. The EPR spectra asserted the geometry around the vanadium center to be a square pyramid for metal complexes. The geometries have been confirmed using DFT under the B3LYP level. Moreover, the quantum parameters proposed promising bioactivity of the oxidovanadium(IV) complexes. The results of the DNA-binding revealed that the investigated complexes bind to DNA via non-covalent mode, and the intrinsic binding constant (K_b_) value for the [VO(SO_4_)(MNZ)_2_] H_2_O complex was promising, which was 2.0 × 10^6^ M^−1^. Additionally, the cytotoxic activity of the synthesized complexes exhibited good inhibition toward both hepatocellular carcinoma (HepG-2) and human breast cancer (HCF-7) cell lines. The results of molecular docking displayed good correlations with experimental cytotoxicity findings. Therefore, these findings suggest that our synthesized complexes can be introduced as effective anticancer agents.

## 1. Introduction

Vanadium (V) is one of the transition metals, with atomic number 23 and composed of two isotopes, ^50^V and ^51^V with atomic abundance of 0.25% and 99.75%, respectively. Vanadium is disseminated across the whole planet in small amounts and is present in the human body in trace amounts [1,2]. The ability of vanadium to influence numerous metabolic processes in the cell is due to possession of the same character traits as phosphate, which plays a vital role in medicinal applications [3]. Therefore, vanadium compounds have been determined in various fields as anticancer, anti-diabetic, anti-parasitic, anti-viral, anti-bacterial, anti-thrombotic, anti-hypertensive, anti-atherosclerotic, spermicidal, anti-human deficiency virus, and anti-tuberculous agents [4,5]. Although there are various drugs for cancer treatment, it remains the second most common cause of death worldwide [6,7]. The limitations of treatment include several side effects, the development of multidrug resistance, and high toxicity, which lead to a critical need to discover novel anticancer compounds with potent anticancer activity, less toxic than the platinum-based drugs, and less side effects [8]. The historical use of oxidovanadium(IV) complexes in various treatments is promising by the addition of new complexes that may be effective against cancer [9,10]. Thus, several studies have reported the cytostatic activity and ability to defeat tumor cell growth in vitro and in vivo of the inorganic compounds of vanadium (valence varied from III to V) coordinated with organic ligands [11].

Imidazole is a five-member ring molecule that has a wide range of biological activities and is an essential pharmacophore in drug discovery [12]. Several imidazole derivatives have been used as effective anticancer agents due to the remarkable pharmaceutical potencies to control cancer [13,14]. One of the significant properties of imidazole is easy binding to DNA or protein molecules via different interactions, such as electrostatic, intercalation, and groove binding [15,16].

In the search, we merged their benefits to obtain novel vanadyl-based drug complexes with a valuable anticancer potency. Three imidazole molecules, named Clotrimazole (CTNZ), Miconazole (MNZ), and Pantoprazole (PNZ), were reacted with oxidovanadium(IV) salt in ethanol solution and then characterized by various approaches. The molecular structures of all new complexes were optimized by theory level DFT/B3LYP to investigate the geometry properties and the quantum parameters. The biological efficiency of the metal complexes was tested by a DNA-binding study and molecular docking. Moreover, an in vitro cytotoxicity assay was carried out against two cell lines, hepatocellular carcinoma (HepG-2) and breast adenocarcinoma (MCF-7).

## 2. Experimental

### 2.1. Materials and Instrumentation

The reagents and chemicals utilized in this research were purchased from various commercial sources with 100% purity (Fisher Scientific, CAD Middle East; Aldrich, Riyadh, Saudi Arabia) and used without purification. Melting points were measured by MPA100, an automated melting point system. The conductivity measurements were determined using a conductor TDA meter at room temperature in 10^−3^ M DMSO solution. IR spectra were recorded using solid samples on a PerkinElmer Spectrum 100 instrument (Waltham, MA, USA), and peaks are reported over the range 4000–400 cm^−1^. The electronic absorption spectra were determined in DMSO from the MultiSpec-1501 UV-Vis spectrophotometer. The EPR spectra for the complexes in solution were recorded at room temperature by a continuous-wave Bruker EMX PLUS spectrometer (Bruker BioSpin, Rheinstetten, Germany) and collected with Bruker Xenon software with DPPH standard (υ = 9.440103 GHz). TGA was obtained from the PerkinElmer TGA system, in the range of 25–900 °C and at a 10 °C min^−1^ heating rate under nitrogen atmosphere.

### 2.2. Synthesis of VO(II) Complexes

Vanadyl sulfate (VOSO_4_.5H_2_O) was dissolved in 3 mL of distilled H_2_O and added dropwise to 25 mL of ethanolic solution of ligand and was refluxed for 2–3 h. The produced precipitates were filtered, collected, washed by hot ethanol, drops of diethyl ether were added, and they were dried in an oven at 50 °C.

**[VO(SO_4_)(CTNZ)(H_2_O)]H_2_O** Yield 74%, m.p. 108 °C. IR (cm^−1^): 3315 ν(OH_2_); 1593 ν(C=N); 1305 ν(C-N); 1276 ν_asym_(SO_4_); 1054 ν_sym_(SO_4_); 751 ν(C-Cl); 973 ν(V=O); 588 ν(V-O); 451 ν(V-N). Elemental analysis of C_22_H_21_ClN_4_O_7_SV (%): Found: C 48.59; H 3.89; N 5.15; Anal. Calc. (%): C 48.51; H 3.80; N 4.99; UV-Vis (1 × 10^–3^ M, DMSO, nm): 259 (π–π∗, strong), 293 (n–π∗, shoulder), 840 (d–d, weak); ᴧm (Ω^−1^ cm^2^ mole^−1^) 11.72; μ_eff_ (BM)1.75.

**[VO(SO_4_)(MNZ)_2_] H_2_O** Yield 91%, m.p. 119 °C. IR (cm^−1^): 3445 ν(OH_2_); 1707 ν(C=N); 1343 ν(C-N); 1289 ν_asym_(SO_4_); 1040 ν_sym_(SO_4_); 1090 ν(C–O–C); 972 ν(V=O); 584 ν(V-O); 438 ν(V-N). Elemental analysis of C_36_H_30_Cl_8_N_4_O_8_SV (%): Found: C 42.67; H 2.98; N 5.53; Anal. Calc. (%): C 42.60; H 2.92; N 5.44; UV-Vis (1 × 10^–3^ M, DMSO, nm): 269 (π–π∗, strong), 304 (n–π∗, shoulder), 830 (d–d, weak); ᴧm (Ω^−1^ cm^2^ mole^−1^) 10.00; μ_eff_ (BM)1.75.

**[VO(PNZ)_2_]SO_4_.2H_2_O** Yield 77%, m.p. 157 °C. IR (cm^−1^): 3442 ν(OH_2_); 1698, 1621 ν(C=N); 1426 ν(C-C); 1027 ν(S=O); 968 ν(V=O); 525 ν(V-O); 448 ν(V-N). Elemental analysis of C_32_H_34_F_4_N_6_O_15_S_3_V (%): Found: C 39.80; H 3.55; N 8.70; Anal. Calc. (%): C 39.67; H 3.49; N 8.76; UV-Vis (1 × 10^–4^ M, DMSO, nm): 264 (π –π∗, medium), 294 (n–π∗, strong), 304 (LMCT, shoulder), 472 (d–d, weak); ᴧm (Ω^−1^ cm^2^ mole^−1^) 10.00; μ_eff_ (BM)113.28.

### 2.3. Molar Ratio Method

The stochiometric ratio of ligands to vanadium in the complexes was determined by the molar ratio method [17,18]. The concentration of metal was kept constant at 0.72 μM and pipetted into seven volumetric flasks (0, 1, 2, 3,…, 6 mL). Then, various ligand concentrations were added in 6, 5, 4, 3,…, 0 mL volumes, as presented in Table S1, Supplementary Materials. All measurements were recorded at 207–291 nm.

### 2.4. Computational Details

Theoretical calculations of the investigated compounds were carried out by the Gaussian 09 program package [19], employing the density function theory method (DFT), and the hybrid density functional B3LYP in the gas phase. The basis set 6-31G was utilized for the free drug molecules while the metal complexes were performed with the correlation-consistent basis set LANL2DZ to accommodate the large size of the target complexes. Additionally, the MEP map was projected by applying the B3LYP/LANL2DZ level within DFT. The vibrational frequencies calculation was obtained by visualizing output files via Gauss View 6.0. All stationary points for optimized molecules are minima on the potential energy surface and do not have imaginary frequencies, and were scaled by factor values of 0.99 and 0.96 to correct for anharmonicity and the neglected aspect of electron correlation.

### 2.5. DNA Interaction Studies

The interaction of oxidovanadium(IV) complexes with Calf Thymus-DNA (CT-DNA, purchased from Sigma Aldrich) was examined in Tris-HCl buffer solution at a pH = 7.4 using UV-Vis absorption spectroscopy. The CT-DNA stock was stored in a refrigerator and used within one week. The solution of CT-DNA was confirmed to be free from proteins by checking the ratio of UV absorbance at 260 and 280 nm (A_260_/A_280_), which was in the 1.8–1.9 range [20,21]. The CT-DNA concentration was determined from the absorbance calculated at λ_max_ 260, with a molar extinction coefficient of 6600 M^−1^ cm^−1^. Stock solutions of oxidovanadium(IV) complexes were dissolved in DMSO (1 × 10^−4^ M) and suitably diluted with the corresponding buffer solution to the required concentrations. The intrinsic binding constant (K_b_) of the complexes was calculated from the plot of [DNA]/(εa − εf) vs. [DNA] using the following equation, where K_b_ is given by the ratio of the slope to the intercept:

$$\left[\mathrm{DNA}\right]/\left(\varepsilon a-\varepsilon f\right)=\left[\mathrm{DNA}\right]/\left(\;\varepsilon b-\varepsilon f\;\right)1+\left[K_{b}\;\left(\varepsilon b-\varepsilon f\right)\;\right]$$

[DNA] is the concentration of CT-DNA, εf is the extinction coefficient of the free compound, εa is equal to A_obsd_/[Compound], and εb is the extinction coefficient of the complexes.

### 2.6. Docking Studies

Molecular docking investigations were performed using the Molecular Operating Environment (MOE) program. The crystal structures of breast cancer (PDB code 1HK7) binding to this protein present the inhibition of hsp90 alpha, which leads to the degradation of client proteins involved in the initiation of breast cancer pathogenesis, and human thymidylate synthase for hepatocellular carcinoma (PDB code 1JU6) proteins were retrieved from the protein data bank (https://www.rcsb.org/ accessed on 30 March 2021). All the water molecules around the proteins were removed, and hydrogen atoms were added. Then, the optimized geometry of each tested compound with the minimum binding energy was attained using the MMFF94x force-field [22,23]. The site finder in the MOE module was used to generate alpha-site spheres. The DFT-optimized structures of the compounds were used to generate the five best binding configurations with flexible molecular rotation. The hydrogen bonds that formed between proteins and the investigated compounds were used to rank the binding affinity, which was expressed as the free binding energy (S, kcal/mol).

The validation of our docking method was performed by docking the co-crystalline ligand with the same protocol and comparing the interaction between it and the downloaded one, and we obtained RMSD in the range of 1.37–1.52 Å.

### 2.7. Anticancer Studies

Oxidovanadium(IV) complexes were evaluated for their anticancer activity against hepatocellular carcinoma (HepG-2) and breast adenocarcinoma (MCF-7). In this assay, a 96-well plate with 100 pl/well was used to plate the cell culture (1 × 10^4^ cells/mL). The tested complex was diluted into each well with calculated concentrations (3.125, 6.25, 12.50, 25.00, 50.00, and 100 ug/mL) and incubated. After 24 h of incubation, MTT ((3-14,5-dimethylthiazol-2-yl)-2,5-diphenylte trazolium bromide) liquid was added and the plate incubated for a further 24 h. Each well’s optical density (OD) was measured spectrophotometrically, and the 50% concentration causing growth inhibitory action (IC_50_ value) to tumor cells was estimated using the following formula:

$$\mathrm{Cell}\;\mathrm{viability}\;\%=\frac{\mathrm{OD}\;\mathrm{value}\;\mathrm{of}\;\mathrm{experimental}\;\mathrm{sample}\;\left(\mathrm{mean}\right)}{\mathrm{OD}\;\mathrm{value}\;\mathrm{of}\;\mathrm{experimental}\;\mathrm{control}\;\left(\mathrm{mean}\right)}\times 100$$

The acute oral toxicity study was performed as documented in [24]. Briefly, healthy BALB/C mice (6–8 weeks) were randomly divided into 7 groups. The [VO(SO_4_)(MNZ)_2_] H_2_O and [VO(CTZ)_2_] 2H_2_O were selected as a result of their good results obtained from molecular docking. After fasting for 4 h, each group (*n* = 6) was administered [VO(SO_4_)(MNZ)_2_] H_2_O or [VO(CTZ)_2_] 2H_2_O as solution in 0.25 mL of DMSO at doses of 2000, 1700, 1400, 1100, 800, 500, and 0 mg kg^−1^ body weight. After vanadium administration, mice were fed normally and observed successively for mortality for 14 days, and the LD_50_ values were calculated using SSPS software. 

Ethical approval was obtained for all animal experiments and was granted by the Unit of Biomedical Ethics Research Committee under registration number HA·02·J·OD8.

## 3. Results and Discussions

### 3.1. Stoichiometry and Physical Properties of the Prepared Complexes

The maximum absorbance wavelengths were measured relative to a blank sample. The absorbance values were then plotted against the ratio of [M]/([M] + [L]) and presented in Figure S1. The reflection line appeared at 0.5 upon increasing the ligand concentration for complex VO-CTNZ, which revealed that the metal:ligand combination ratio was 1:1. In contrast, the reflection line for complexes VO-MNZ and VO-PNZ was around 0.33, indicating a 1:2 M:L ratio.

The physical properties and the elemental analysis for the ligands and their complexes are listed in Table 1. All complexes are insoluble in water and most traditional organic solvents, but soluble in dimethylformamide (DMF) and dimethylsulfoxide (DMSO). Molar conductivity measurements of the obtained complexes were performed at 25 °C in DMSO at a concentration of 10^−3^ M. The molar conductivity values for complexes VO-CTNZ and VO-MNZ ranged between 11.72 and 3.00 Ω^−1^ cm^2^ mol^−1^, respectively, thus revealing their non-electrolytic nature. Complex VO-PNZ afforded higher values, indicating the presence of sulfate outside the coordination sphere.

**Figure 1:**
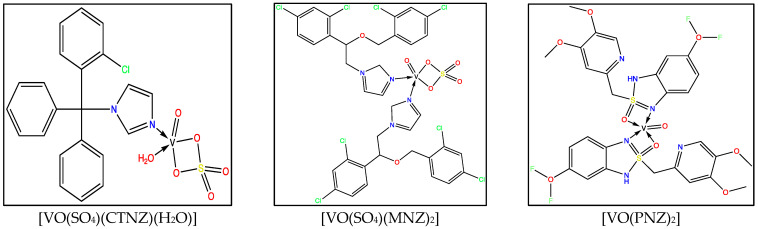
Suggested structures of synthesized oxidovanadium(IV) complexes.

The suggested structures of the complexes were established by CHN analysis, various spectroscopic methods, and thermal analysis. They displayed a square pyramidal geometry and were stable toward air and moisture (Figure 1).

### 3.2. Theoretical Studies

#### 3.2.1. Optimized Molecular Geometry

The results from the spectroscopic analysis (IR, UV-Vis, and EPR) were used to generate the input file to proceed with a computational study and to optimize the complexes’ geometry in gas phase using the DFT method. The extracted optimized geometries from the log file are presented with the number and label atoms in Table S2. The comparison between the free ligand and the formed complexes showed elongation or compression after the reaction with metal ions. The bond lengths around the metal coordination for PNZ, C18-S19, C20-S19, and S19-O31 were longer than the free ligand, except C18-S19 was shorter with 0.04 Å A. The bond angles also showed a practical change after complexation (Table 2), where the bond angles decreased by 0.009–0.038° due to the electric repulsion after complexing. On the other hand, the changes in the MNZ and CTNZ were negligible after the reaction with oxidovanadium(IV) for both bond lengths and angles.

The new bonds between oxidovanadium(IV) and O and N sites in all investigated compounds had average bond lengths of 1.09–2.09, which are assumed to have an ionic character of the metal to oxygen and nitrogen, respectively [25], excluding the bond length of V-O45 in the CTNZ, which showed a covalent bond character with bond length >2 Å. Moreover, the angles around the individual central vanadium ion indicated that the compounds implemented a pyramidal geometry, as presented in Table S2, Supplementary Materials. The dihedral angles around the metal ions were between 175° and −175°, which are away from 0° or 180°, indicating that the ligand molecules and metal ions are not on the same plane.

#### 3.2.2. Assignments of Vibrational Spectra

Vibration frequency analysis is an essential tool to investigate the binding coordination mode for metal complexes through peak shift observation [26]. The IR spectra were measured between 4000 and 400 cm^−1^ for the ligands and their oxidovanadium(IV) complexes. Moreover, the FT-IR analysis for the free and metal compounds was performed with DFT/B3LYP in the same range. The essential functional groups and their assignments were compared with theoretical calculation and listed in Table 3.

The IR spectra of the ligands showed a characteristic peak between 1590 and 1494 cm^–1^ for the imidazole ν(C=N) group, which was shifted to higher frequencies in all complexes in the range of 1622–1593 cm^–1^, confirming the coordination of imidazole nitrogen to the central vanadium ion. Further, the IR spectrum of free PNZ showed a band at 1043 cm^−1^ assigned to ν(S=O) of the side chain. This band appearing at 1027 cm^−1^ in VO-PNZ indicated the coordination of the O atom to the vanadium ion [27]. The broad peaks in the region from 3445 to 3315 cm^−1^ are due to the stretching vibrations of water molecules, ν(OH_2_). Furthermore, new bands observed in the spectrum of complex VO-CTNZ at 874 and 799 cm^−1^ which might be assigned to δ_r_(H_2_O) at 874 cm^−1^ and δ_w_(H_2_O) at 799 cm^−1^ affirmed coordination with the water molecule [28]. The bands at 1091 and 1070 cm^−1^ in the spectrum of the free MNZ and PNZ drugs, respectively, corresponding to the stretching vibrations of the C–O–C group have shifted in the obtained coordination compounds to 1090 cm^−1^ for complex VO-MNZ and to 1068 cm^−1^ for complex VO-PNZ. Additionally, the bands relating to ν(SO_4_) in the range 1289–1040 cm^−1^ support its bidentate coordination [29]. In all VO(II) complexes, a significant ν(V=O) band was recorded around ≈970 cm^−1^. There were new bands that appeared in the complexes’ spectra in the regions 588–525 and 451–438 cm^−1^, which were attributed to the formation of ν(V-O) and ν(V-N) bonds, respectively. The calculated frequencies were well-connected with the experimental results.

**Figure 2:**
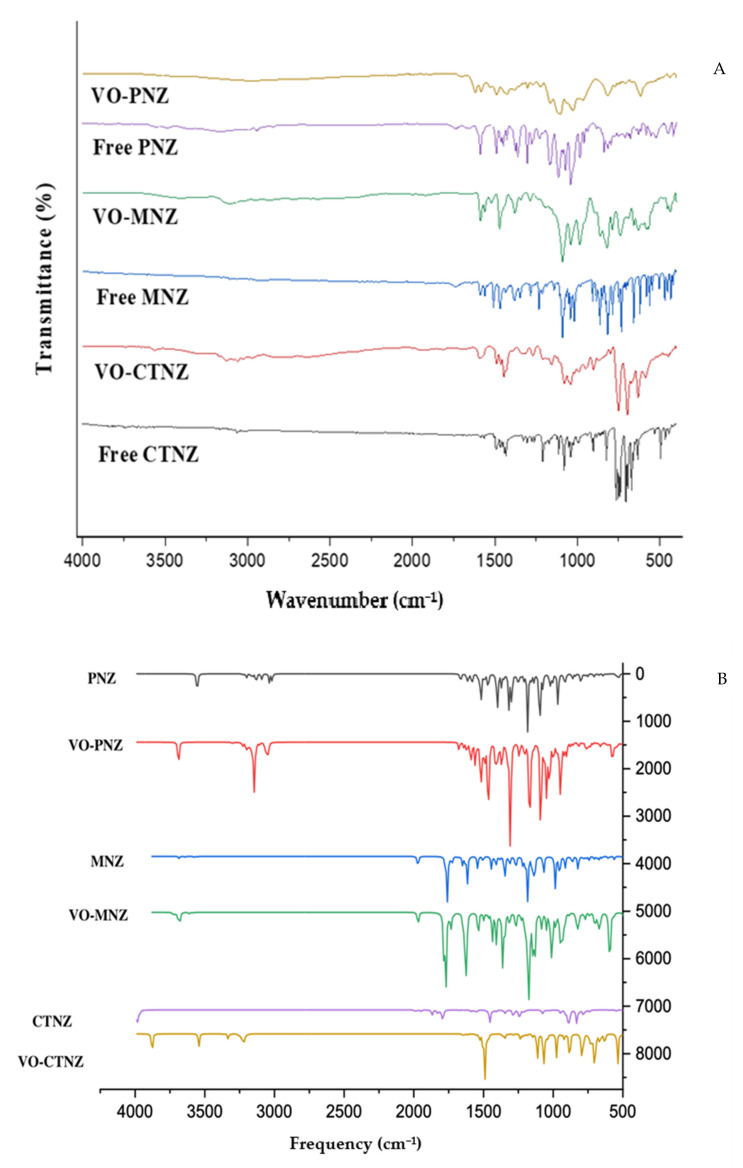
A comparison of the experimental (**A**) and theoretical (**B**) using DFT/ B3LYP IR spectra for the free ligands and the oxidovanadium(IV) complexes.

The metal-oxygen bond length (V=O) was determined from the stretching frequencies and calculated by the below equation [30,31]:

v = 21,349 exp(−1.9176 R (Å)),

where the value of v is the V=O stretching frequency in cm^−1^, and R assignment is the V=O bond length in angstroms. The calculated bond lengths for [VO(SO_4_)(CTNZ)(H_2_O)], [VO(SO_4_)(MNZ)_2_], and [VO(PNZ)_2_]SO_4_ complexes were 1.611, 1.611, and 1.613 Å, respectively. From Table 3, a real correlation between the experimental results with the obtained theoretical values can be seen, 1.60–1.59 Å. Figure 2 shows the comparison of the theoretical and experimental values of the IR spectra assignments of the free ligands and the synthesized metal complexes.

#### 3.2.3. Frontier Molecular Orbitals (FMOs) Parameters

**Figure 3:**
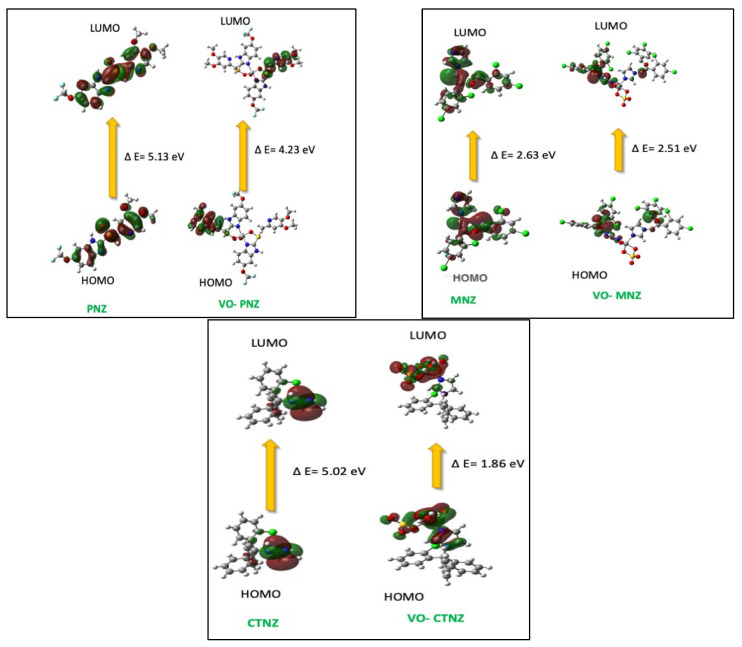
HOMO and LUMO charge density maps of the studied complexes using the B3LYP/LAND.

A practical method to describe the electronic characteristics of molecular arrangements and study the orbital compositions is the frontier molecular orbitals (FMO) [32]. These orbitals were generated from the fchk files and visualization for both the highest occupied molecular orbital (HOMO) and lowest unoccupied molecular orbital (LUMO) to study the molecular orbital compositions and energy levels of a molecule, and are presented in Figure 3. The ground state, HOMO, for the PNZ was mainly distributed over most of the ligand molecule, while it was concentrated in one of the dimethoxypyridin-2-yl methanethiols in the metal complex, while the LUMO was at the other one in the ligand molecule of the ratio 2:1.

In contrast, MNZ showed a distribution of HOMO and LUMO over one of the coordination ligand molecules. The HOMO for CTNZ was located on the metal center and the donating atoms, which raise the ability of the metal complex to react as a donor or an accepter to react with the biological residue. The obtained values for the HOMO and LUMO for the metal complexes and the free organic molecules have been used to calculate the energy gap, E_g_ = E_LUMO_ − E_HOMO_. This indicator is usually used to assess the reactivity of the molecules [33]. A small E_g_ value indicates the reactivity of the complexes through to the softness character, while the large energy gap between HOMO and LUMO complicates the transition of the electron, and we can express this as hard compounds. Table 4 shows that the E_g_ values were decreased after coordination to the metal ion, and CTNZ had the smallest value among the investigated compounds and was thus the most reactive molecule. For more details of the molecules’ properties, we estimated the global reactivity descriptors, which are absolute electronegativities (χ), absolute hardness (η), chemical potentials (μ), global softness (S), and global electrophilicity (ω), using equations in our previous work [33] and illustrated in Table 4. The absolute softness is the reverse of the absolute hardness, which describes the sense to react with other molecules such as biological compounds, and we could arrange them according to the absolute softness (S) as follows: VO-CTNZ > VO-MNZ > VO-PNZ. Thus, VO-CTNZ complex had the maximum S value, indicating high anticipation of effective biological activity. The electrophilicity indicator (χ) has a positive value, but the electronic chemical potential (μ) has a negative value, which revealed the capability of the new complexes to capture electrons from the environment to decrease their energy and to stabilize the reaction. Therefore, the following order presents the most potent compound with a high electrophilicity indicator: VO-CTNZ > VO-MNZ > MNZ >VO-PNZ > CTNZ > PNZ.

#### 3.2.4. Molecular Electrostatic Potential Maps (MEPs)

**Figure 4:**
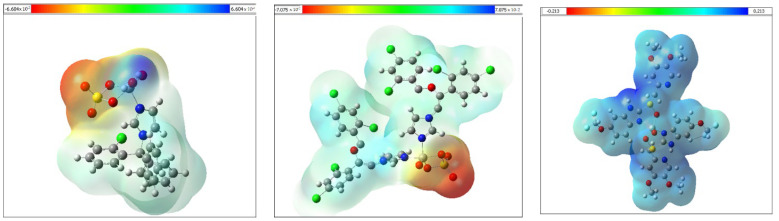
The molecular electrostatic potential surface of the metal complexes calculated by the DFT/B3LYP level.

The calculated MEPs were obtained to provide the molecule electron distribution over the compounds, which provided insight into the electrophilic and nucleophilic attack sites. Molecular electrostatic potential surface maps of the metal complexes are presented in Figure 4. The maps have a red color which presents the electron-rich zone and the nucleophile site, while the blue areas represent the positive electrophile region. The VO-CTNZ and VO-MNZ showed that the SO_4_ group had the highest electronic density in the molecules, while the rest of the molecules were mostly in the blue area and reacted as electrophile. Interestingly, the blue color was distributed over the whole of the VO-PNZ complex, which increased the tendency of the molecule toward electrophilic attack with the negative phosphate group in the biological residues.

### 3.3. Electronic Spectroscopic and Magnetic Susceptibility

**Figure 5:**
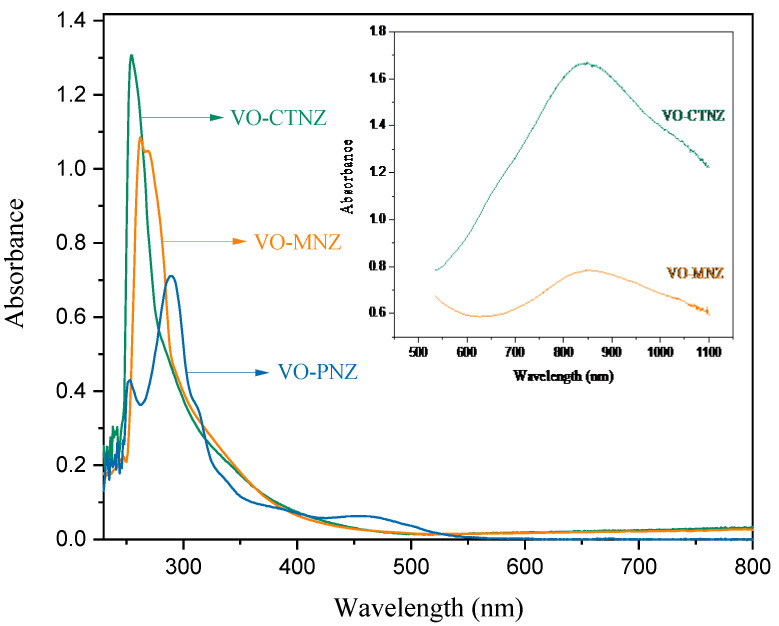
The UV-Vis spectra of synthesized oxidovanadium(IV) complexes in DMSO.

Electronic spectral data of all the ligands and metal complexes are listed in Table 5 and the spectra are shown in Figure 5, measured in DMSO solution (10^−^^3^ to 10^−4^ M) in the range of 200 to 1000 nm at room temperature. The complexes contain bands in the ranges of 257–273 and 280–299 nm, which are ascribed to π–π* transitions that originate mainly from the aromatic rings and n→π* transitions that could be assigned to the C=N chromophore. These bands shifted to higher wavelengths after the reaction with metal ions.

Low-intensity peaks observed at higher wavelengths around 800 nm in VO-CTNZ and VO-MNZ are due to low-energy d–d transitions [34]. However, the electronic spectrum of VO-PNZ shows a shoulder band at 317 nm and a low-intensity band at 472 nm that may be attributed to LMCT, and the spin-allowed d–d electronic transitions, respectively [34].

Moreover, the effective magnetic moment values (μ_eff_) were calculated from the measured magnetic susceptibility using the following equation:

µ_eff_ = 2.828 (ꭕ_m_T)^1/2^ BM

where T is the absolute temperature and ꭕ_m_ is the molar susceptibility constant [35,36]. Complexes VO-CTNZ, VO-MNZ, and VO-PNZ had magnetic moment values of 1.75, 1.75, and 1.76 Bohr’s magneton (BM), respectively. The obtained values indicated that the VO(II) complexes have normal paramagnetic properties, with one unpaired electron and square pyramidal geometry around the central metal ion [37,38].

### 3.4. EPR Spectroscopy

**Figure 6:**
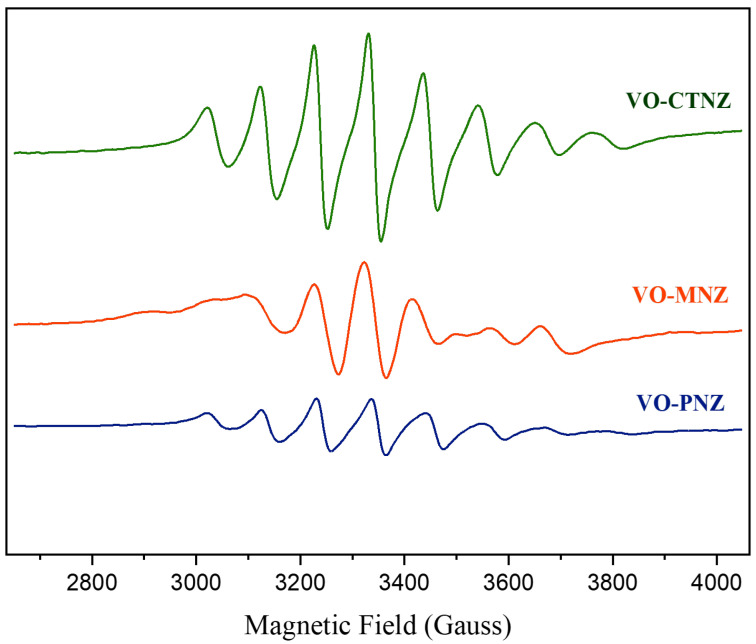
EPR spectra of oxidovanadium(IV) complexes.

EPR spectra of the VO(II) complexes were recorded in DMF at ambient temperature, and the spectral parameters are summarized in Table 6. The spectra exhibit eight lines, which are due to hyperfine splitting arising from the interaction of the unpaired electron with a ^51^V nucleus having the nuclear spin I = 7/2 (Figure 6). This confirms the presence of a single cation as the metallic center in the complex. The measured values of g and A are in good agreement for a square pyramidal structure [39,40].

### 3.5. Thermal Analysis

**Figure 7:**
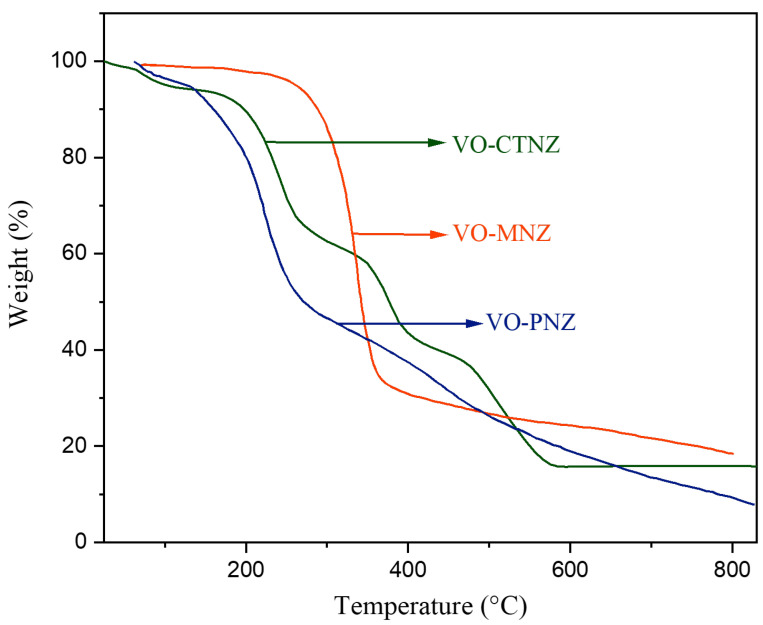
The curves of TG for three oxidovanadium(IV) complexes.

The thermogravimetric analysis (TGA) was measured from 25 to 900 °C to confirm the stability of our complexes and the molecular structure (Figure 7), and the results were summarized in Table S3. The TG curve for [VO(SO_4_)(CTNZ)(H_2_O)] H_2_O complex proceeded via four degradation steps. The first step of decomposition occurred within the range of 25–105 °C, with a total mass loss of 3.29% (calc. 3.14%), corresponding to the loss of one water molecule of hydration. The second step of decomposition occurred in the temperature range of 105–278 °C, accompanied by a weight loss of 30.32% (calc. 30.70%), and was attributed to the loss of one coordinated water molecule and C_6_H_6_N_2_Cl. The third stage, in the temperature range of 278–442 °C, is assigned to the loss of C_10_H_6_O with a mass loss of 25.85% (calc. 25.70%). The final step of decomposition occurred at 442–650 °C and was accompanied by a weight loss of 25.26% (calc. 25.37%), corresponding to the loss of C_6_H_2_O_2_S, affording VO_2_ as a final product with a mass loss of 15.27% (calc. 15.25%).

[VO(SO_4_)(MNZ)_2_] H_2_O complex was thermally decomposed in one step within the temperature range from 25 to 800 °C, accompanied by a weight loss of 80.35% (calc. 80.98%), and corresponding to the loss of C_27_H_28_Cl_8_N_4_O_5_S and half-hydrated water molecules, forming VO_2_ and 9 carbon atoms as a final product with a mass loss of 19.65% (calc. 19.01%).

The decomposition of [VO(PNZ)_2_]SO_4_·2H_2_O occurred in two steps. The first step was within a temperature range of 25–109 °C and corresponds to the loss of two moles of hydrated water with a mass loss of 3.88% (calc. 3.73%). The second step was within a temperature range of 109–800 °C and equals the loss of the organic moiety, C_32_H_30_F_4_N_6_O_11_S_3_, with a weight loss of 87.59% (calc. 87.68%). The final product is VO_2_ residue, with a total mass loss of 8.52% (calc. 8.59%).

### 3.6. Kinetic Studies of the TGA

Recently, the Coats–Redfern (CR) [41] and Horowitz–Metzger (HZ) [42] methods have been used effectively around the world to depict thermal decomposition kinetics. The entropy (ΔS), enthalpy (∆H), and Gibbs free energy (ΔG) were calculated using previous equations [43]. According to the kinetic parameters presented in Table S4, Supplementary Materials, for each step of decomposition, large positive values of E_a_ indicate that the complexes are extremely stable [9,44]. The values of ΔS for the complexes were negative, which indicates that the thermal decomposition was slower than the formation reaction, or that there was a greater degree of balance within the activated complex than in the reactants [45]. The positive ΔH values suggest that all the decomposition steps were endothermic in nature. The high values of ΔG for each stage are due to the increase in TΔS values from one step to the next. Moreover, the correlation coefficient values demonstrate that the two methods used provide a good linear function for the thermal decomposition stages of the complexes. The Coats–Redfern and Horowitz–Metzger plots of all complexes are represented in Table S5, Supplementary Materials.

### 3.7. Biological Application

#### 3.7.1. DNA-Binding Study

**Figure 8:**
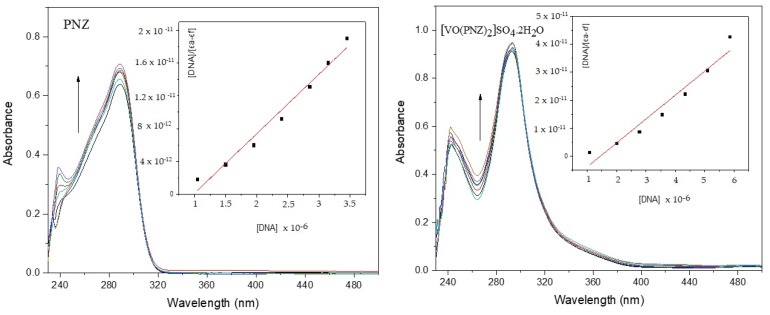
Absorption spectra of PNZ and its complex in the presence of increasing DNA concentration.

The electronic absorption spectra were evaluated for the ligands and their oxidovanadium(IV) complexes by monitoring the changes in the maximum absorption bands in the range 262–301 nm assigned to the π→π∗ transitions of the aromatic rings (Figure 8 and Figure S3). The UV-Vis absorbance of the free ligands and VO-PNZ showed increasing molar absorptivity (hyperchromic) with increasing CT-DNA concentration. Hyperchromism occurs due to the breakage of the secondary structure of DNA after the interaction with compounds and DNA, which derogates the interaction forces maintaining the DNA double-helix structure. Thus, the solution had many bases in free helix form and did not form hydrogen bonds with complementary bases, causing heightened absorbance [46,47]. The obtained results clearly show a groove-binding mode of binding of the free ligands and VO-PNZ complex to DNA. On the other hand, the absorption bands of VO-CTNZ and VO-MNZ decreased (hypochromic) with the rising amount of CT-DNA. Hypochromism is an intercalative reaction between the aromatic part of the compound with the base-pair π electrons of the DNA, resulting in a decrease of the distance between this and the π electron in the aromatic ring [48]. On further addition, there was a notable blue shift with hypochromism, indicating that VO-CTNZ and VO-MNZ have potentially used intercalation to bond with the DNA. The determined binding constants (K_b_) for the complexes were ordered as follows: VO-MNZ > VO-PNZ > VO-CTNZ. The calculated K_b_ and absorption data of the complexes are presented in Table S6, Supplementary Materials.

#### 3.7.2. Molecular Docking

Molecular docking helps us to understand the possible modes of interaction and binding affinities of new compounds as targeted drug molecules, which may offer better insight into how well they exert bioactivity against the target [45]. The docked complex conformations were evaluated according to the docking scores and binding interactions of the ligands and oxidovanadium(IV) complexes with the hepatocellular carcinoma (ID: 1JU6) and breast cancer (ID: 1HK7) proteins, which were selected according to the literature and previous studies [49]. The docking study results indicated that the ligands and synthesized complexes involve non-covalent interactions, such as π–cation, π–π, ionic, and hydrogen bonding interactions, as presented in Table S7. All the studied compounds exhibited excellent docking score values with the proteins (1JU6 and 1HK7). In general, the more negative the energy value (S), the greater the stability and the stronger the binding between the docking sites and protein receptors. The binding free energy docking scores (S) and root-mean-square deviations (RMSD) of atomic positions are presented in Tables S8 and S9, Supplementary Materials. The estimated binding scores of the free ligands CTNZ, MNZ, and PNZ were calculated as −6.66, −8.09, and −8.59 kcal/mol, respectively, for the 1JU6 protein, while the binding score values of the synthesized complexes VO-CTNZ, VO-MNZ, and VO-PNZ were −7.23, −11.09, and −11.30 kcal/mol, respectively. The score values against the 1JU6 protein toward the free ligand were −6.81, −7.11, and −7.11 kcal/mol, respectively, and −9.85, −7.64, and −11.10 kcal/mol, respectively, for their metal complexes. Notably, the results revealed high docking scores for the complexes compared to those for the free ligands. This indicates that the modified metal complexes enhance the binding affinity of the free ligands. In terms of the coordination pattern, the VO-CTNZ complex formed a π–π bond and four hydrogen bonds with the following amino acid residues: oxygen 45 with CYS 195, oxygen 46 with HIS 256, oxygen 48 with ARG 50, oxygen 49 with SER 216, and the benzene ring with TRP 109, with bond distances of 2.80–3.53 Å. In the 1HK7 protein, the VO-CTNZ complex formed H-acceptor bonds via oxygen 44 and oxygen 49 atoms with TRP 109 and ARG 344 amino acid residues, with bond distances of 3.00–3.42 Å. Additionally, in the 1JU6 protein, the main contributions to the activity of the VO-MNZ complex were the interactions with the amino acids CYS 195, ARG 50, ASN 112, ASP 218, SER 216, and LEU through hydrogen and ionic bonds, with bond distances of 3.10–4.11 Å. Moreover, the imidazole ring displayed π–π bonding with the PHE 140 amino acid residue, and π–H bonding with the ARG 51 amino acid residue in the 1HK7 protein, with bond distances of 3.73–3.80 Å. The VO-PNZ complex displayed the best binding scores of the docked complexes against both proteins (−11.30 and −11.10 kcal/mol), forming four hydrogen bonds with ARG 78, MET 311, ILE 108, and PHE 225 amino acids for the 1JU6 protein, with bond distances of 3.39–4.40 Å. An additional H-acceptor bond was observed between VO-PNZ and TYR 337 in the 1HK7 protein, with a bond distance of 2.63 Å. All the 3D and 2D interactions for the two targets are illustrated in Table 7 and Table S10.

#### 3.7.3. Cytotoxicity Study

**Figure 9:**
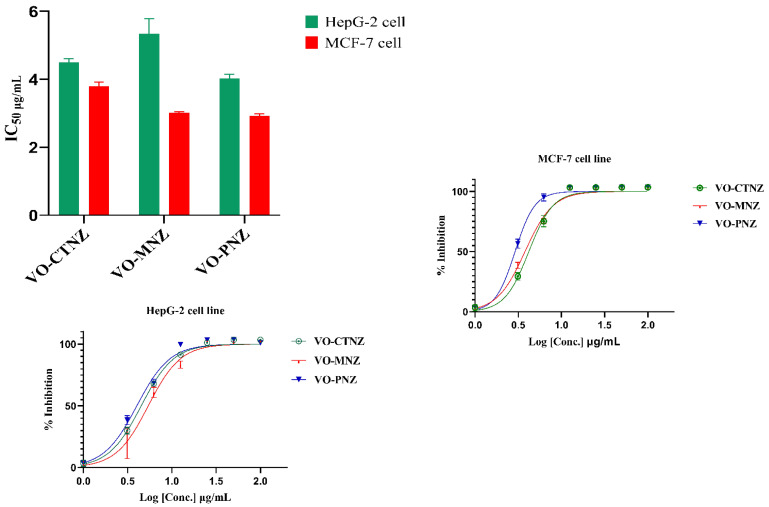
Inhibition and IC_50_ of selected compounds in DMSO solutions toward HepG-2 and MCF-7 cell lines.

**Figure 10:**
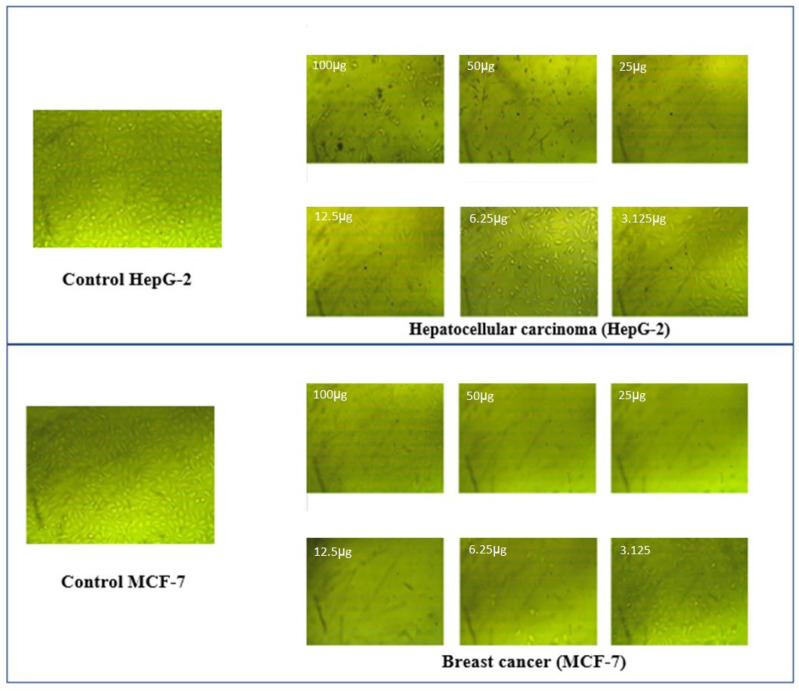
Morphology images of hepatocellular carcinoma (HepG-2) and breast cancer (MCF-7) treatment by [VO(PNZ)_2_]SO_4_·2H_2_O.

Our aim in this study was to evaluate the anticancer activity of newly synthesized oxidovanadium(IV) complexes against two different human tumor cell lines: HepG-2 and MCF-7 cells. We have chosen MCF-7 and HepG-2 cells because they are considered the most common types of carcinomas. The calculated IC_50_ values are shown in Table 8 and a graphical statistical analysis is represented in Figure 9. Their performance was compared to an anticancer chemotherapy drug, cisplatin, as a standard reference. Initially, the results were consistent with the docking predictions, revealing that all the synthesized vanadyl complexes displayed excellent activities against these two cell lines. The IC_50_ (μg/mL) values indicate the following order of toxicity towards the HepG-2 cell line: VO-PNZ > VO-CTNZ > VO-MN. According to the results obtained for the HCF-7 cell line, the order of toxicity is as follows: VO-PNZ > VO-MNZ > VO-CTNZ. The molar concentration (μM) showed that the VO-PNZ potency is double the inhibition of VO-CTNZ toward HepG-2. Despite that VO-PNZ has a good effect against the HCF-7 cell line, with 2.93 μg/mL, the obtained μM value of VO-MNZ was better, with 0.05 μM. Morphological observations of the HepG-2 and MCF-7 cell lines showed noteworthy changes in their morphology and decreased cell density, as shown in Figure 10, Figures S4 and S5. Interestingly, all metal complexes showed better inhibition toward HepG-2 than cisplatin, and the difference between the IC_50_ values in the case of MCF-7 for [VO(PNZ)_2_]SO_4_·2H_2_O and cisplatin was 0.50 μg/mL. Moreover, the oral LD_50_ of [VO(SO_4_)(MNZ)_2_] H_2_O and [VO(CTZ)_2_] 2H_2_O was calculated to be 1235.92 and 944.66 mg kg^−1^, respectively. According to Loomis and Hayes’ classification [50], a chemical substance with an LD_50_ within the range of 5000–15,000 mg kg^−1^ is regarded as practically nontoxic, slightly toxic, moderately toxic, and highly toxic, respectively. The results indicated that both complexes fall within the category indicating a slightly toxic substance. Based on the results, it may be concluded that the synthesized oxidovanadium(IV) complexes exhibit promising anticancer activity against both cell lines [51].

## 4. Conclusions

New oxidovanadium(IV) complexes were synthesized and characterized by various techniques. From the elemental analysis, electronic spectra, magnetic moment values, molar conductance, and EPR, the newly synthesized complexes were found to have a square pyramidal geometry around the central metal ion, with a molar ratio of the metal to the ligand of 1:1 for VO-CTNZ, and a 1:2 (M:L) ratio for VO-MNZ and VO-PNZ. The DFT study confirmed this geometry structure and reveled that the complexes were more reactive than the free ligand. The VO-CTNZ showed the highest softness among the prepared complexes, which could indicate favorable biological activity. The positive charge around the VO-PNZ presented by MPE indicated a practical tendency to react toward biological molecules. A kinetic study was conducted of the thermal decomposition using the Coats–Redfern and Horowitz–Metzger methods, suggesting that all complexes were non-spontaneous, and thereby that the complexes were thermally stable. DNA-binding features were investigated by using spectrophotometric titrations. The binding ability of the mentioned complexes followed the order: VO-MNZ > VO-PNZ > VO-CTNZ. Anticancer activity was tested for all compounds against hepatocellular carcinoma and human breast cancer cell lines, in which all complexes exhibited a significant inhibition, with the highest cytotoxicity value for the VO-PNZ. Additionally, the in vitro cytotoxicity of compounds was supported by docking studies.

## Figures and Tables

**Table 1:** Analytical and physical data of ligands and their oxidovanadium(IV) complexes.

Complex	M.wt. (g mol^−1^)	Color	Yield %	M.P. (°C)	% Found (Calc.)			µeff. (B.M.)	Λm (Ω^−1^ mol^−1^cm^2^)
					C	H	N		
CTNZ	344.9	White	–	140	76.60 (76.56)	4.90 (4.91)	8.10 (8.07)	–	0
[VO(SO_4_)(CTNZ)(H_2_O)]H_2_O	543.9	Dark green	74	108	48.59 (48.51)	3.89 (3.80)	5.15 (4.19)	1.75	11.72
MNZ	416.1	White	–	80	51.96 (51.97)	3.39 (3.34)	6.73 (6.75)	–	0
[VO(SO_4_)(MNZ)_2_] H_2_O	1013.3	Olive green	91	119	42.67 (42.60)	2.98 (2.92)	5.53 (5.57)	1.75	3.00
PNZ	383.4	Beige	–	158	50.13 (50.11)	3.94 (3.94)	10.96 (10.95)	–	0
[VO(PNZ)_2_]SO_4_.2H_2_O	965.8	Orange	77	167	39.80 (39.67)	3.55 (3.49)	8.70 (8.76)	1.76	113.28

**Table 2:** Selected geometric bond lengths, bond angles, and dihedral angles of the optimized ligand and oxidovanadium(IV) complexes.

Bond Lengths			Bond Angles			Dihedral Angles		
	PNZ	VO-PNZ		PNZ	VO-PNZ		PNZ	VO-PNZ
C18-N15	1.31	1.35	N15-C18-S19	128.6	119.1	N15-C18-S19-O31	178.71	−175.71
C18-S19	1.85	1.81	C18-S19-O31	97.7	93.9	C21-C20-S19-O31	77.61	−130.31
C20-S19	1.72	2.80	C20-S19-O31	102.1	84.81	N57-C60-S61-O73	-	19.9
S19-O31	1.98	1.77	N57-V-N15	-	155.33	C21-C20-S19-O31	-	−175.7
V-N15	-	2.07	O31-V-O73	-	138.24	S61-O73-V-O84	-	−70.21
V-O31	-	1.93	N15-V-O31	-	83.30	S19-O31-V-O84	-	−79.03
V-N57	-	1.09	N57-V-O73	-	82.35	C18-N15-V-O84	-	95.4
V-O73	-	2.00	N15-V-O84	-	102.20	C20-S19-V-O84	-	−87.19
V=O84	-	1.59	O31-V-O84	-	110.10	S61-O73-V-O84	-	−70.21
**Bond Lengths**			**Bond Angles**			**Dihedral Angles**		
	**MNZ**	**VO-MNZ**		**MNZ**	**VO-MNZ**		**MNZ**	**VO-MNZ**
C17-N22	1.40	1.41	C17-N22-C16	105.61	107.50	C13-N21-C16-N22	178.45	174.95
C16-N22	1.33	1.33	N22-V-N56	-	90.72	C16-N22-V-O72	-	114.17
V-N22	-	2.09	O71-V-O79	-	77.18	C17-N22-V-O72	-	−61.90
V-O71	-	1.96	O72-V-N22	-	106.10	C50-N56-V-O72	-	6.19
V-N56	-	2.08	O72-V-N56	-	97.74	C51-N56-V-O72	-	−164.66
V-O79	-	1.94	O72-V-O71	-	114.40	S73-O71-V-O72	-	113.17
V=O72	-	1.60	O72-V-O79	-	118	S73-O70-V-O72	-	−109
**Bond Lengths**			**Bond Angles**			**Dihedral Angles**		
	**CTNZ**	**VO-CTNZ**		**CTNZ**	**VO-CTNZ**		**CTNZ**	**VO-CTNZ**
C2-N8	1.39	1.40	C2-N8-C3	105.40	107.06	C2-N8-C3-N4	111.80	109.80
C3-N8	1.33	1.35	N8-V-O45	-	85	C2-N8-V-O47	-	−15.01
V-N8	-	2.03	N8-V-O47	-	99.27	C2-N8-V-O46	-	−112.39
V=O47	-	1.60	N8-V-O46	-	105.14	S50-O45-V-O46	-	133.73
V-O45	-	2.28	N8-V-O44	-	116.81	S50-O45-V-O47	-	136.51
V-O44	-	1.84	O45-V-O46	-	65.73	S50-O44-V-O47	-	−168.63
V-O46 (H_2_O)	-	2.04	O45-V-O47	-	94.60	S50-O44-V-O46	-	−54.98

**Table 3:** The main bands in IR spectra for ligands and their oxidovanadium(IV) complexes.

Compound	ν(C=N)		ν(S=O)		ν_asym_ and ν_sym_ (SO_4_)		ν(V=O)		ν(V-O)		ν(V-N)	
	Exp.	DFT	Exp.	DFT	Exp.	DFT	Exp.	DFT	Exp.	DFT	Exp.	DFT
CTNZ	1494	1487	-		-		-		-		-	
[VO(SO_4_)(CTNZ)(H_2_O)] H_2_O	1593	1492	-		1276–1054	1250–1026	973	946	588	515	451	423
MNZ	1509	1471	-		-		-		-		-	
[VO(SO_4_)(MNZ)_2_] H_2_O	1522	1505	-		1289–1040	938–849	972	1039	584	549	438	441
PNZ	1590	1588	1043	1033	-		-		-		-	
[VO(PNZ)_2_]SO_4_.2H_2_O	1622	1538	1027	1026	-	-	968	1058	525	515	448	377

**Table 4:** The calculated quantum chemical parameters of the ligands and their oxidovanadium(IV) complexes.

Compound	HUMO	LUMO	∆E	χ	η	σ	μ	S	ω	ΔN _max_
CTNZ	−6.84	−1.82	5.02	4.33	2.51	0.40	−4.33	1.25	3.74	1.73
VO-CTNZ	−10.80	−8.95	1.86	9.88	0.93	1.08	−9.88	0.46	4.94	10.63
MNZ	−6.25	−3.62	2.63	4.93	1.31	0.76	−4.93	0.66	2.47	3.75
VO-MNZ	−6.72	−4.21	2.51	5.47	1.25	0.80	−5.47	0.63	2.73	4.36
PNZ	−6.24	−1.11	5.13	3.67	2.57	0.39	−3.67	1.28	1.84	1.43
VO-PNZ	−6.61	−2.38	4.23	4.49	2.12	0.47	−4.49	1.06	2.25	2.12

**Table 5:** Electronic spectral data of the studied compounds.

Compound	π–π*	n–π*	d–d
	nm		
CTNZ	262	272	-
[VO(SO_4_)(CTNZ)(H_2_O)] H_2_O	259	293	~840
MNZ	273	280	-
[VO(SO_4_)(MNZ)_2_] H_2_O	269	304	~830
PNZ	257	299	-
[VO(PNZ)_2_] SO_4_·2H_2_O	264	294	472

**Table 6:** EPR parameters of oxidovanadium(IV) complexes.

Complex	g	A
[VO(SO_4_)(CTNZ)(H_2_O)] H_2_O	1.995	110
[VO(SO_4_)(MNZ)_2_] H_2_O	1.996	100
[VO(PNZ)_2_]SO_4_·2H_2_O	1.983	107

**Table 7:** 2D and 3D docking interactions of the studied complexes with colon cancer protein “Human Thymidylate Synthase” for hepatocellular carcinoma protein (PDB code = 1JU6) and breast cancer (PDB code = 1HK7) protein.

Types of Protein	Compound	2D Snapshot	3D Snapshot
Hepatocellular carcinoma	PNZ	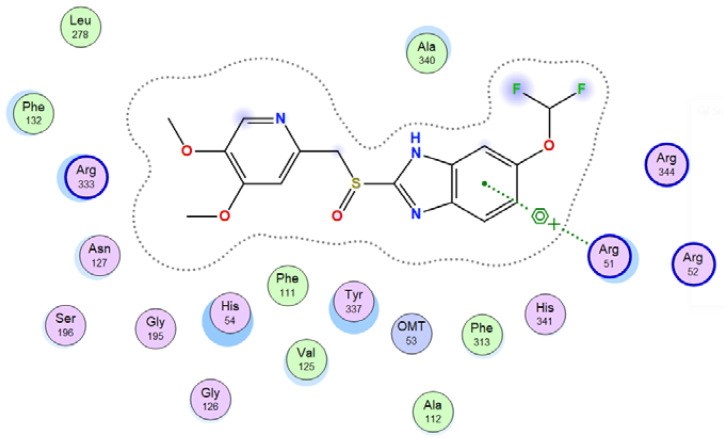	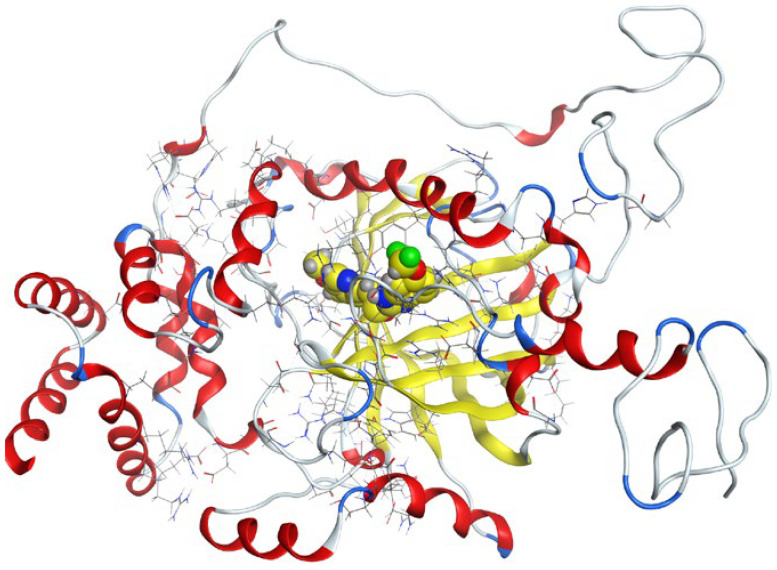
	VO-PNZ	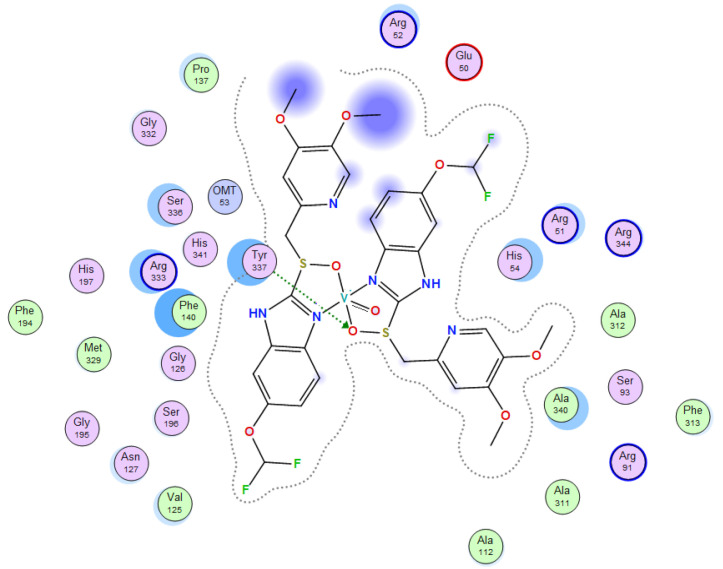	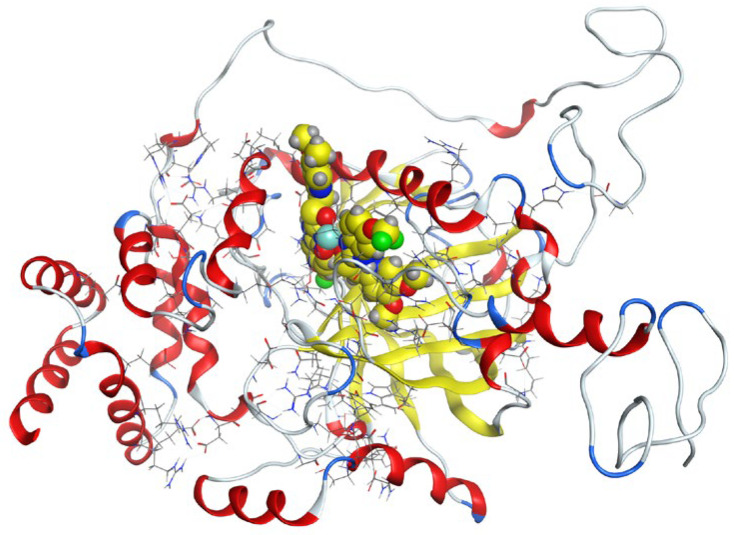
Breast cancer	PNZ	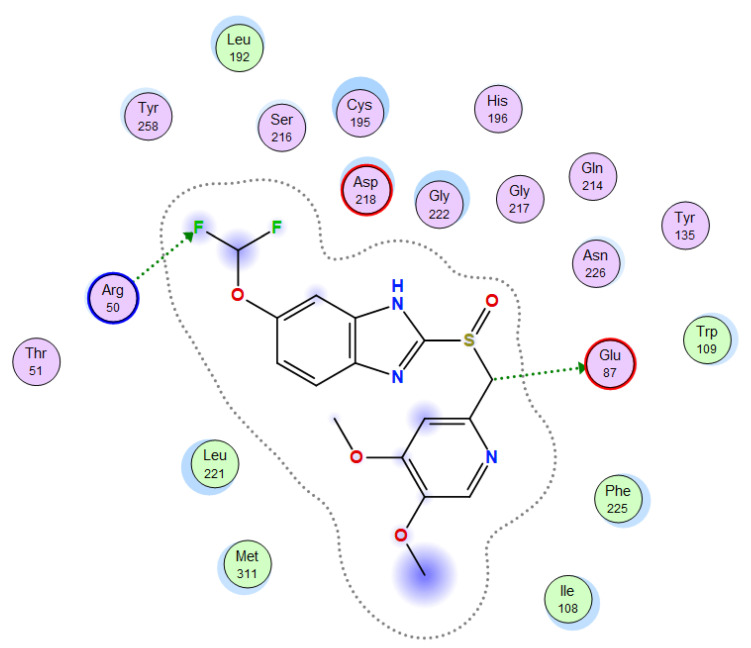	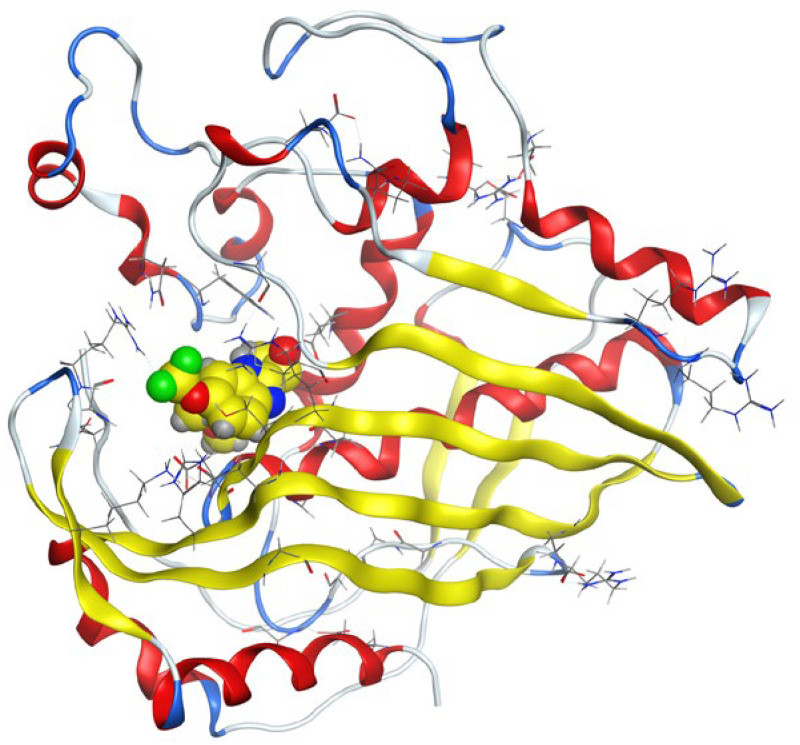
	VO-PNZ	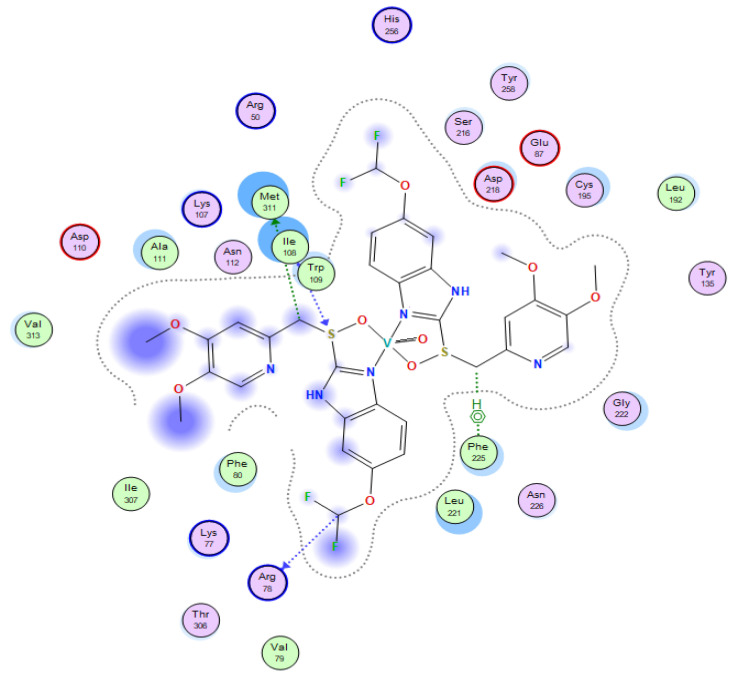	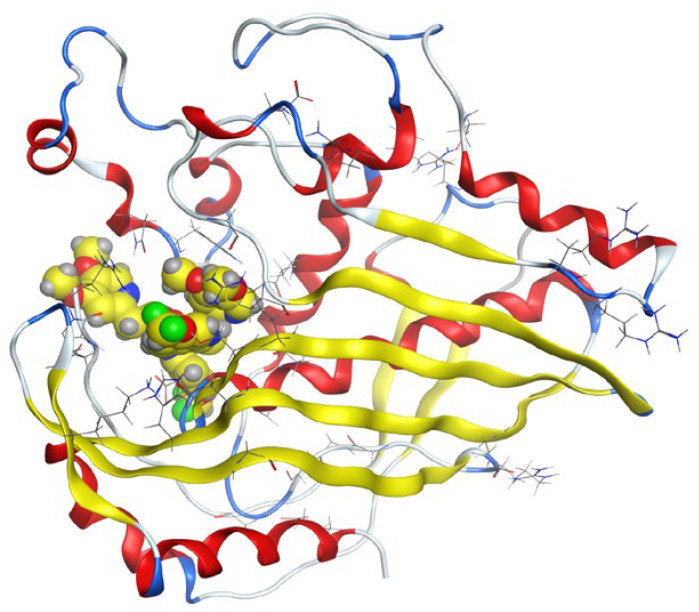
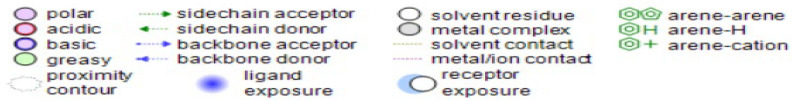			

**Table 8:** IC_50_ for tested compounds against different cell lines compared to cisplatin in different units, IC_50_ ± SD (μg/mL) and μM.

Complex	HepG-2		MCF-7	
	μg/mL	μM	μg/mL	μM
[VO(SO_4_)(CTNZ)(H_2_O)]H_2_O	4.5 ± 0.11	8.27	3.79 ± 0.13	6.97
[VO(SO_4_)(MNZ)_2_]H_2_O	5.34 ± 0.45	5.27	3.02 ± 0.02	2.98
[VO(PNZ)_2_]SO_4_·2H_2_O	4.02 ± 0.13	4.16	2.93 ± 0.06	3.03
Cisplatin	25.5 [52]		2.43 [25]	

IC_50_: 1–10 (very strong), 11–20 (strong), 21–50 (moderate), 51–100 (weak), and above 100 (non-cytotoxic).

## Data Availability

All relevant data are within the manuscript and its Supplementary Materials.

## Supplementary Materials

The following supporting information can be downloaded at: https://www.mdpi.com/article/10.3390/molecules27092796/s1, Table S1: Experimental data by molar ratio method; Figure S1: The molar ratio for VO(II) complexes; Table S2: The optimized geometry and numbering system for the free ligands and Oxidovanadium(IV) complexes; Table S4: Kinetic parameters of thermal decomposition steps for VO(II) complexes; Table S5: Coats-Redfern (CR) and Horowitz–Metzger (HM) of the VO(II) complexes; Figure S2: The UV-Vis spectra of free ligands; Figure S3: Absorption spectra of free ligands and their complex in the presence of increasing DNA concentration; Table S6: The spectral parameters for the interaction of VO(IV) complexes and their ligands with DNA; Table S7: interaction table between the compounds and 1JU6 “Human Thymidylate Synthase” for Hepatocellular carcinoma protein and Breast cancer (1HK7) proteins; Table S8: Docking score and energy of the compounds and 1JU6 “Human Thymidylate Synthase” for Hepatocellular carcinoma protein; Table S9: Docking score and energy of the compounds and 1Hk7 Breast cancer protein; Table S10: 2D and 3D Docking interaction of the VO(II) complexes with colon cancer protein “Human Thymidylate Synthase” for Hepatocellular carcinoma protein (PDB code = 1JU6) and Breast cancer (PDB code = 1HK7) proteins; Figure S4: Morphology images of Hepatocellular carcinoma (HepG-2) treatment by [VO(SO_4_)(CTNZ)(H_2_O)]H_2_O and [VO(SO_4_)(MNZ)_2_] H_2_O; Figure S5: Morphology images of breast cancer (MCF-7) treatment by [VO(SO_4_)(CTNZ)(H_2_O)]H_2_O and [VO(SO_4_)(MNZ)_2_] H_2_O.

## Sample Availability

Samples of the investigated compounds are available from the authors.
