# Supplementary material for: Theoretical Investigation by DFT and Molecular Docking of Synthesized Oxidovanadium(IV)-Based Imidazole Drug Complexes as Promising Anticancer Agents
Source: Molecules. 2022 Apr 27;27(9):2796. doi: 10.3390/molecules27092796 (PMC9105665; doi:10.3390/molecules27092796)
Supplement: Supplementary file 1 [file molecules-27-02796-s001.zip › molecules-1673732-supplementary.pdf]

Supplementary Materials

Theoretical Investigation by DFT and Molecular Docking of Synthesized Oxidovanadium(IV)-Based Imidazole Drug Complexes as Promising Anticancer Agents

Amal S. Basaleh [1], Fatimah Y. Alomari [2], Abeer A. Sharfalddin [1], Najlaa S. Al-Radadi [3], Doaa Domyati [4] and Mostafa A. Hussien [1,5,*]

[1] Department of Chemistry, Faculty of Science, King Abdulaziz University, P.O. Box 80203 Jeddah 21589, Saudi Arabia.
[2] Chemistry Department, College of Science, Imam Abdulrahman Bin Faisal University, P.O. Box 76971, Dammam 31441, Saudi Arabia.
[3] Department of Chemistry, Faculty of Science, Taibah University, P.O. Box 30002, Al-Madinah Al-Munawarah 14177, Saudi Arabia.
[4] Department of Chemistry, College of Science, University of Jeddah, P.O. BOX 80327, Jeddah 21589, Saudi Arabia.
[5] Department of Chemistry, Faculty of Science, Port Said University, Port Said 42521, Egypt.
* Correspondence: maabdulaal@kau.edu.sa

Table S1. Experimental data by molar ratio method.

NO.	Metal conc. (×10^{-4} moles)	Ligand conc. (×10^{-4} moles)	[M]/([M]+[L])	$[VO(SO_4)(CTNZ)(H_2O)]\ H_2O$ at 211 nm	$[VO(SO_4)(MNZ)_2]H_2O$ at 207 nm	$[VO(PNZ)_2]SO_4.2H_2O$ at 213 nm
**1**	0.72	2.52	0.22	1.53	1.00	1.17
**2**	0.72	2.16	0.25	1.36	0.89	1.03
**3**	0.72	1.80	0.28	1.29	0.82	0.89
**4**	0.72	1.44	0.33	1.19	0.70	0.93
**5**	0.72	1.08	0.40	1.08	0.63	0.71
**6**	0.72	0.72	0.50	0.95	0.61	0.72
**7**	0.72	0.36	0.60	0.83	0.53	0.53
**8**	0.72	0.00	1.00	0.74	0.43	0.44

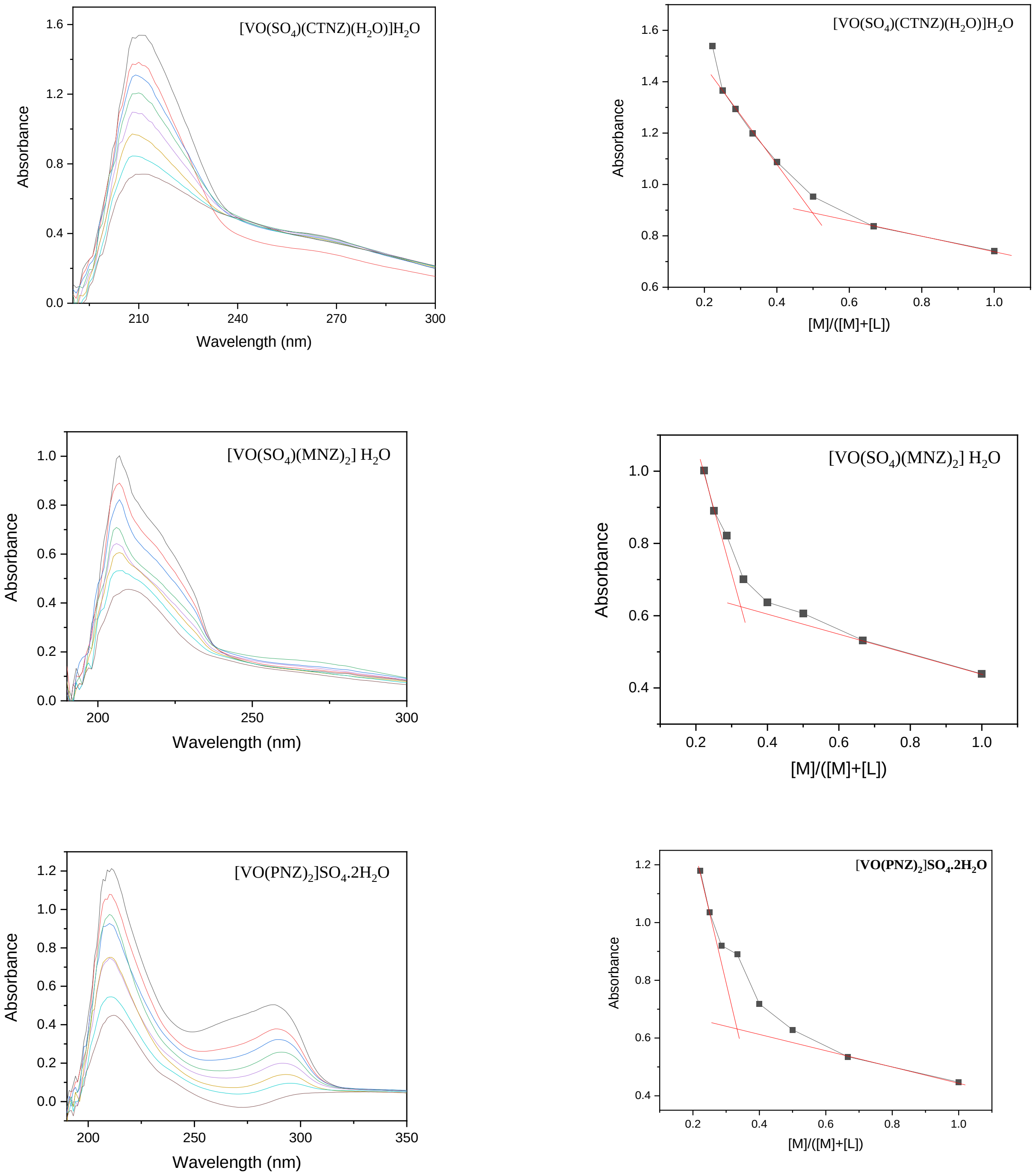

Figure S1. The molar ratio for VO(II) complexes.

Table S2. The optimized geometry and numbering system for the free ligands and Oxidovanadium(IV) complexes.

Ligand	Complex

PNZ	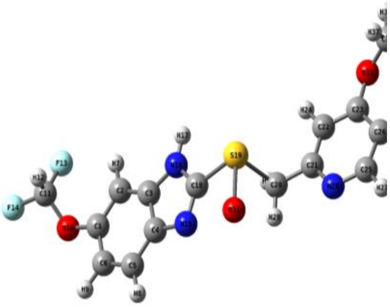	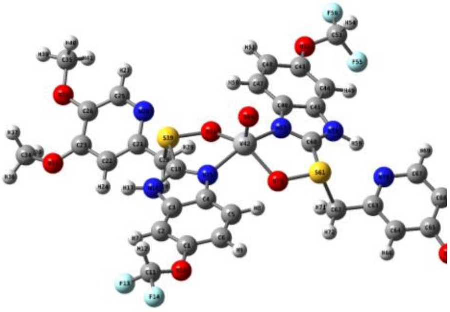
MNZ	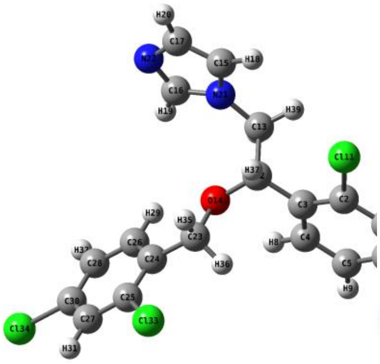	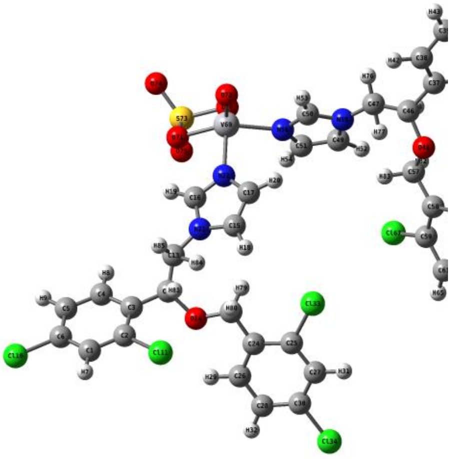
CTNZ	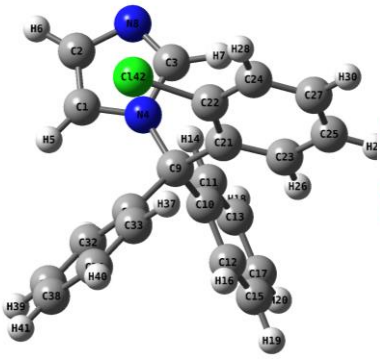	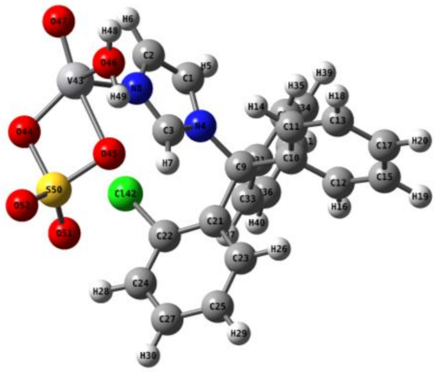

Table S3. Thermogravimetric analysis data for the VO(II) complexes.

Complex	Step	Temp. range (°C)	Weight loss % found (calc)	Assignments	Total mass loss/% found (calc)	Final solid-state residue %
$[VO(SO_4)(CTNZ)(H_2O)]\ H_2O$	1st	25–105	3.29 (3.14)	H_2O	84.72 (85.17)	VO_2 15.27(15.25)
	2nd	105–278	30.32 (30.70)	$H_2O + C_6H_6ClN_2$		
	3rd	278–442	25.85 (25.70)	$C_{10}H_6O$		
	4th	442–650	25.26 (25.37)	$C_6H_2O_2S$		
$[VO(SO_4)(MNZ)_2]\ H_2O$	1st	25–800	80.35 (80.98)	$C_{27}H_{28}Cl_8N_4O_5S$	80.35 (80.98)	VO_2 19.65 (19.01)
$[VO(PNZ)_2]\ SO_4.2H_2O$	1st	25–109	3.88 (3.73)	$2H_2O$	91.47 (91.41)	VO_2 8.52 (8.59)
	2nd	109–800	87.59 (87.68)	$C_{32}H_{30}F_4N_6O_{11}S_3$		

Table S4. Kinetic parameters of thermal decomposition steps for VO(II) complexes.

Complex	Temp. range C°	Method	Ea (kJmol-1)	A (s^{-1})	ΔS (J mol^{-1} K^{-1})	ΔH ($kJmol^{-1}$)	ΔG (kJ mol^{-1})	Correlation coefficient (R)
[$VO(SO_4)(CTNZ)(H_2O)$] H_2O	25–105	CR	6.07×10^4	1.23×10^7	-1.10×10^2	5.80×10^4	9.35×10^4	0.99
		HZ	6.12×10^4	9.19×10^7	-9.31×10	5.85×10^4	8.86×10^4	0.99
		Average	6.10×10^4	5.21×10^7	-1.01×10^2	5.83×10^4	9.11×10^4	0.99
	105-278	CR	6.79×10^4	5.10×10^4	-1.59×10^2	6.39×10^4	1.39×10^5	0.99
		HZ	7.61×10^4	1.56×10^6	-1.30×10^2	7.21×10^4	1.34×10^5	0.99
		Average	7.20×10^4	8.07×10^5	-1.44×10^2	6.80×10^4	1.37×10^5	0.99
	278-442	CR	1.10×10^5	4.00×10^6	-1.25×10^2	1.05×10^5	1.82×10^5	0.99
		HZ	1.20×10^5	7.98×10^7	-9.98×10	1.15×10^5	1.77×10^5	0.99
		Average	1.15×10^5	4.19×10^7	-1.12×10^2	1.10×10^5	1.80×10^5	0.99
	442-650	CR	1.05×10^5	2.38×10^4	-1.69×10^2	9.85×10^4	2.22×10^5	0.99
		HZ	1.17×10^5	1.03×10^6	-1.37×10^2	1.11×10^5	2.11×10^5	0.99
		Average	1.11×10^5	5.26×10^5	-1.53×10^2	1.05×10^5	2.17×10^5	0.99
[$VO(SO_4)(MNZ)_2$] H_2O	25-800	CR	1.03×10^5	2.72×10^6	-1.28×10^2	9.80×10^4	1.75×10^5	0.99
		HZ	1.13×10^5	4.03×10^7	-1.05×10^2	1.08×10^5	1.72×10^5	0.99
		Average	1.08×10^5	2.15×10^7	-1.16×10^2	1.03×10^5	1.73×10^5	0.99
[$VO(PNZ)_2$] $SO_4.2H_2O$	25-109	CR	3.33×10^4	5.29×10^2	-1.94×10^2	3.04×10^4	9.78×10^4	0.99
		HZ	3.88×10^4	4.31×10^3	-1.76×10^2	3.60×10^4	9.71×10^4	0.99
		Average	3.61×10^4	2.52×10^3	-1.85×10^2	3.32×10^4	9.74×10^4	0.99
	109-800	CR	4.19×10^3	4.44×10^4	-3.17×10^2	1.86×10^3	2.28×10^5	0.99
		HZ	1.63×10^4	9.16×10^3	-2.91×10^2	1.03×10^4	2.22×10^5	0.99
		Average	1.02×10^4	4.80×10^3	-3.04×10^2	6.08×10	2.25×10^5	0.99

Table S5. Coats-Redfern (CR) and Horowitz–Metzger (HM) of the VO(II) complexes.

Complex	Stage	Coats-Redfern (CR)	Horowitz–Metzger (HM)
[VO(SO$_4$)(CTNZ)(H$_2$O)] H$_2$O	25–105	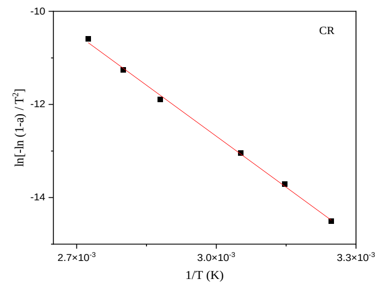	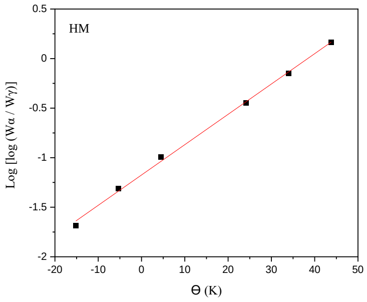
	105-278	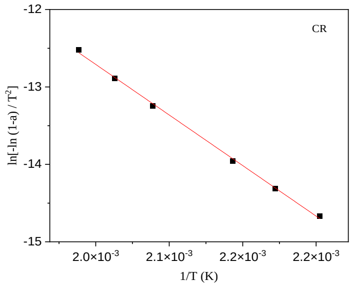	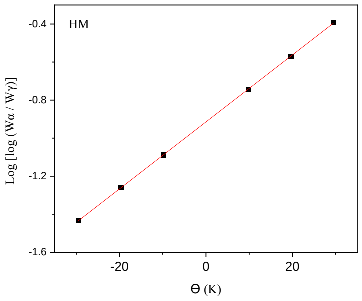
	278-442	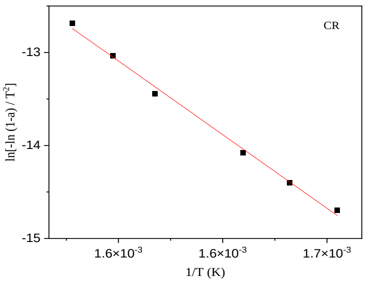	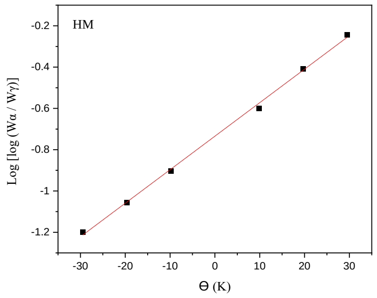
	442-650	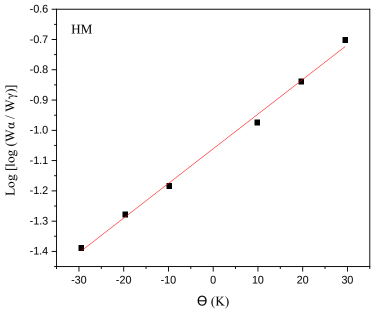	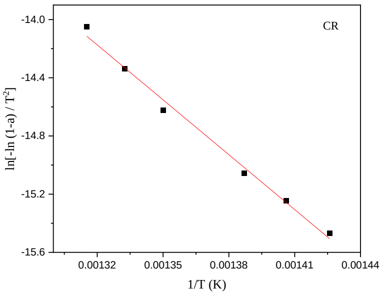

Complex	Stage	Coats-Redfern (CR)	Horowitz–Metzger (HM)
$[VO(SO_4)(MNZ)_2]\ H_2O$	25-800	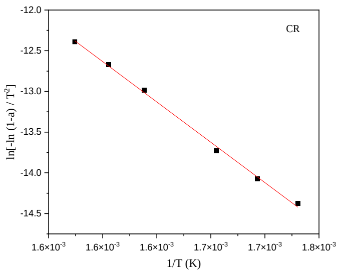	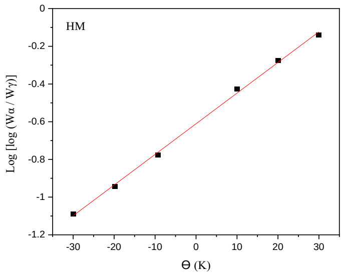
$[VO(PNZ)_2]\ SO_4.2H_2O$	25-109	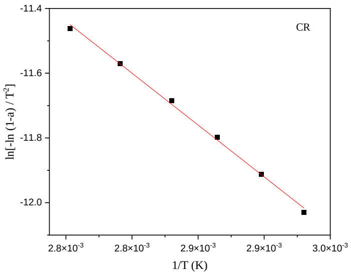	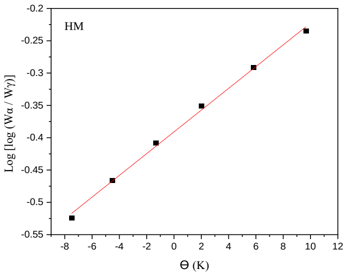
	109-800	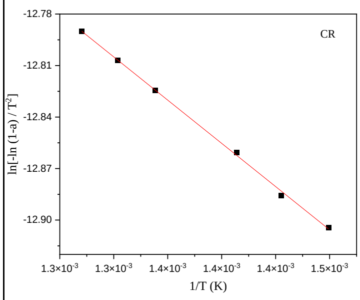	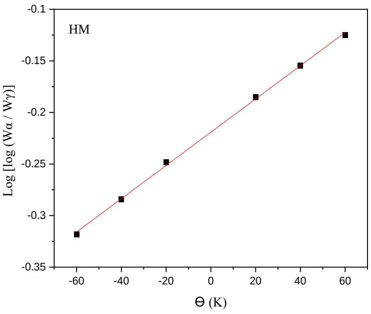

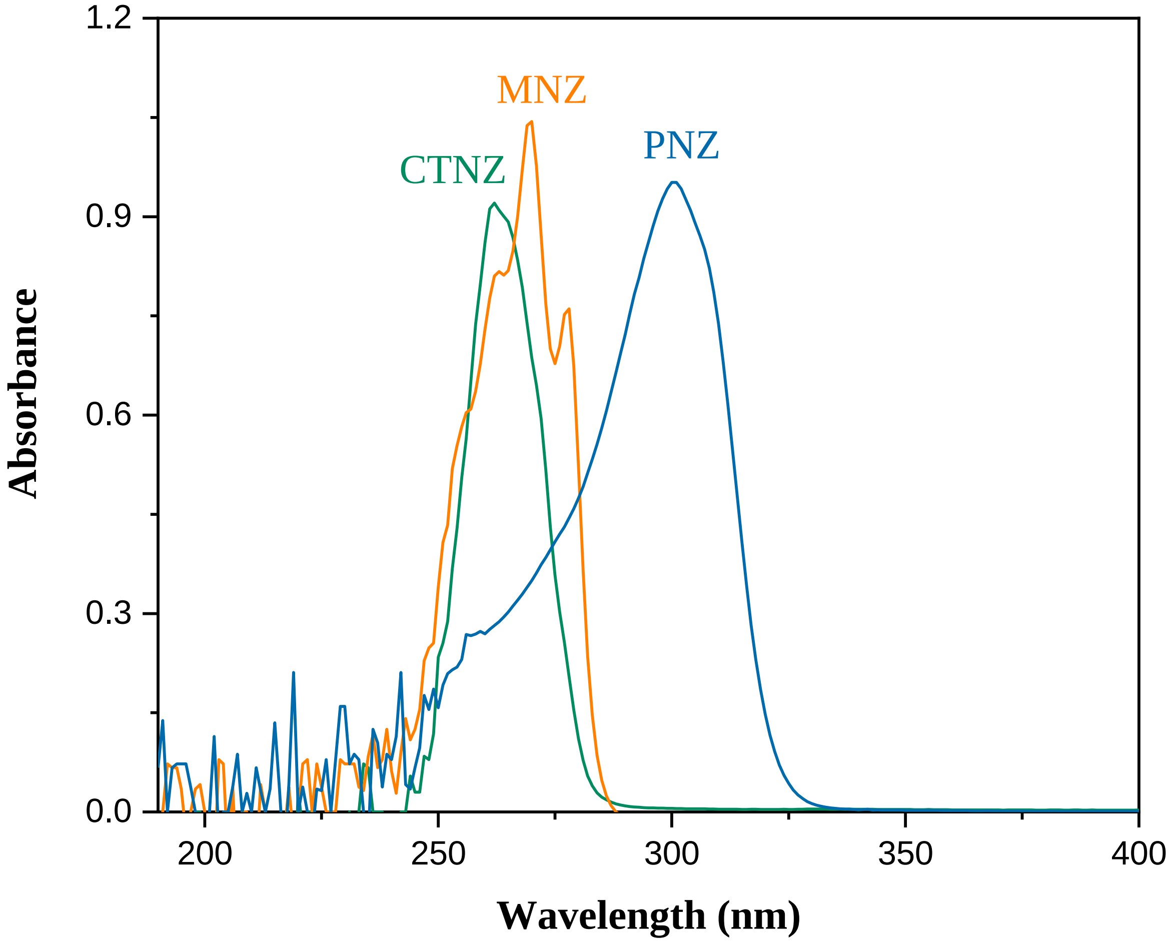

Figure S2. The UV.vis spectra of free ligands.

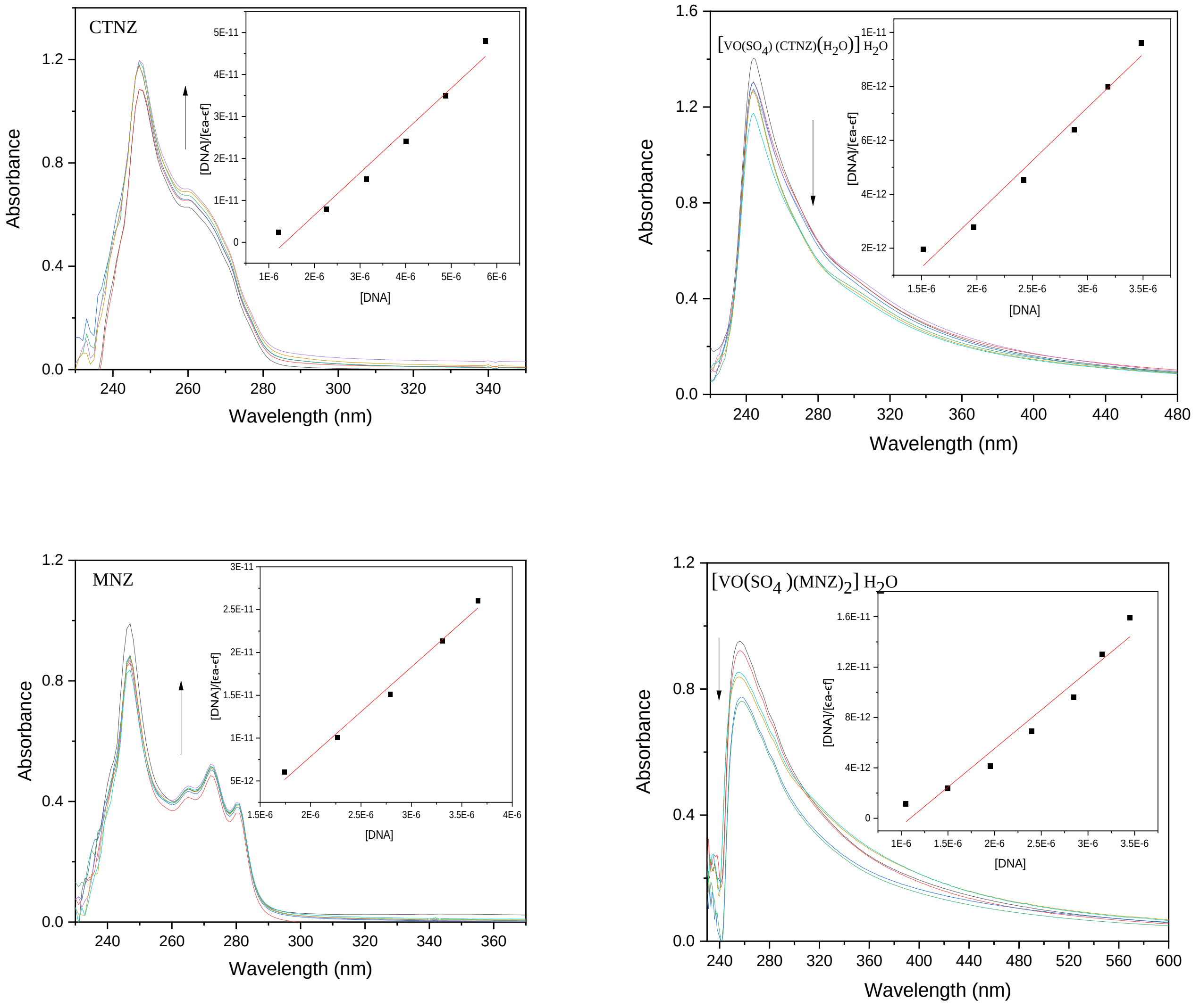

Figure S3. Absorption spectra of free ligands and their complex in the presence of increasing DNA concentration.

Table S6. The spectral parameters for the interaction of VO(IV) complexes and their ligands with DNA.

Compound	K_b (M^{-1})	λ_{max} free (nm)	λ_{max} bound (nm)	Type of chromism
CTNZ	1.00 × 10^6	262	260	Hyperchromic
$[VO(SO_4)(CTNZ)(H_2O)]H_2O$	6.67 ×10^5	262	244	Hypochromic
MNZ	1.00 ×10^6	273	273	Hyperchromic
$[VO(SO_4)(MNZ)_2]$ H_2O	2.00 ×10^6	272	256	Hypochromic
PNZ	1.60 ×10^6	301	289	Hyperchromic
$[VO(PNZ)_2]SO_4.2H_2O$	9.00 ×10^5	295	293	Hyperchromic

Table S7. interaction table between the compounds and 1JU6 "Human Thymidylate Synthase" for Hepatocellular carcinoma protein and Breast cancer (1HK7) proteins.

Types of Protein	Compound	Ligand	Receptor	Interaction	Distance	E(kcal/mol)
Hepatocellular carcinoma	CTNZ	6-ring	6-ring TRP 109 (A)	π - π	3.83	-0.0
	VO-CTNZ	O 45	SG CYS 195 (A)	H-donor	3.07	-8.6
		O 46	CE1 HIS 256 (A)	H-acceptor	3.24	-1.0
		O 48	NH1 ARG 50 (A)	H-acceptor	2.80	-3.8
		O 49	OG SER 216 (A)	H-acceptor	3.03	-2.0
		6-ring	6-ring TRP 109 (A)	π - π	3.53	-0.0
	MNZ	C 12	SD MET 311 (A)	H-donor	3.82	-0.6
		CL 38	O ALA 312 (A)	H-donor	3.46	-1.3
		CL 39	OG SER 216 (A)	H-donor	3.16	-1.1
		5-ring	6-ring TRP 109 (A)	π - π	3.57	-0.0
	VO-MNZ	C 80	SG CYS 195 (A)	H-donor	4.06	-1.8
		O 6	NH2 ARG 50 (A)	H-acceptor	3.10	-1.8
		O 6	ND2 ASN 112 (A)	H-acceptor	3.18	-0.9
		O 4	NH1 ARG 50 (A)	Ionic	3.22	-3.1
		N 14	OD1 ASP 218 (A)	Ionic	3.69	-1.3
		N 30	OD1 ASP 218 (A)	Ionic	3.96	-0.6
		5-ring	OG SER 216 (A)	π -H	3.62	-1.5
		6-ring	CB LEU 221 (A)	π -H	4.11	-0.6
	PNZ	C 39	OE2 GLU 87 (A)	H-donor	3.53	-0.6
		F 32	NH1 ARG 50 (A)	H-acceptor	3.15	-0.5
	VO-PNZ	C 29	O ARG 78 (A)	H-donor	3.39	-1.5
		C 39	SD MET 311 (A)	H-donor	4.18	-0.7
		S 37	CA ILE 108 (A)	H-acceptor	3.64	-0.8
		C 80	6-ring PHE 225 (A)	H- π	4.40	-0.7
Breast cancer	CTNZ	C 35	OD1 ASN 127 (A)	H-donor	3.43	-0.5
		5-ring	ND2 ASN 127 (A)	π -H	3.85	-1.2
		6-ring	5-ring HIS 54 (A)	π - π	3.60	-0.0
	VO-CTNZ	O 44	OH TYR 337 (A)	H-acceptor	3.42	-0.8
		O 49	NE ARG 344 (A)	H-acceptor	3.00	-5.2
		O 49	NH2 ARG 344 (A)	H-acceptor	3.37	-0.9
	MNZ	6-ring	5-ring HIS 54 (A)	π - π	3.44	-0.0
		6-ring	6-ring PHE 140 (A)	π - π	3.63	-0.0
	VO-MNZ	6-ring	CB ARG 51 (A)	π -H	3.78	-0.6
		5-ring	6-ring PHE 140 (A)	π - π	3.80	-0.0
	PNZ	6-ring	NE ARG 51 (A)	π -cation	3.65	-0.5
	VO-PNZ	O 79	OH TYR 337 (A)	H-acceptor	2.63	-2.6

Table S8. Docking score and energy of the compounds and 1JU6 "Human Thymidylate Synthase" for Hepatocellular carcinoma protein.

Compound	S	rmsd_refine	E_conf	E_place	E_refine	E_score2

CTNZ	-6.81	1.60	112.90	-72.46	-24.81	-6.81
	-6.52	1.10	118.91	-84.39	-20.36	-6.52
	-6.52	2.10	105.72	-70.09	-20.37	-6.52
	-6.45	1.54	114.31	-74.76	-23.46	-6.45
	-6.44	1.66	108.02	-71.60	-29.57	-6.44
VO-CTNZ	-9.85	1.62	-759.87	-32.07	7.52	-9.85
	-9.46	2.02	-791.12	-113.45	-26.39	-9.46
	-9.20	1.94	-750.79	-44.65	54.91	-9.20
	-8.36	2.80	-605.88	0.35	45.89	-8.36
	-8.20	2.96	-729.15	-95.11	-37.29	-8.20
MNZ	-7.11	1.03	10.91	-78.23	-39.72	-7.11
	-6.83	1.08	10.35	-107.08	-27.96	-6.83
	-6.80	2.05	12.57	-68.76	-42.92	-6.80
	-6.75	2.40	13.73	-68.99	-30.36	-6.75
	-6.69	1.36	17.41	-91.53	-38.55	-6.69
VO-MNZ	-7.64	1.71	-738.12	-79.33	-7.41	-7.64
	-7.36	1.23	-743.32	-111.77	-21.49	-7.36
	-7.34	3.06	-716.34	-73.51	-19.11	-7.34
	-7.29	1.67	-715.78	-78.61	-12.69	-7.29
	-7.21	2.38	-732.57	-72.33	-10.79	-7.21
PNZ	-7.11	1.53	33.30	-101.60	-39.54	-7.11
	-6.91	2.77	28.28	-82.70	-32.68	-6.91
	-6.89	2.05	39.68	-77.52	-30.31	-6.89
	-6.87	2.19	28.78	-67.93	-32.06	-6.87
	-6.83	1.27	36.24	-74.84	-34.67	-6.83
VO-PNZ	-11.10	2.13	-340.16	-73.37	-43.21	-11.10
	-10.47	2.35	-342.15	-33.13	-55.64	-10.47
	-10.21	2.89	-294.07	-42.67	-49.96	-10.21
	-10.13	2.92	-301.33	-31.44	-34.40	-10.13
	-10.11	2.20	-276.86	-37.16	-30.05	-10.11

where, S= Final score, which is the score of the last stage that was not set to none. rmsd_refine= The root means square deviation between the pose before refinement and the pose after refinement. E_conf = The energy of the conformer. If there is a refinement stage, this is the energy calculated at the end of the refinement. Note that for Forcefield refinement, by default, this energy is calculated with the solvation option set to Born. E_place = Score from the place-ment stage. E_refine = Score from the refinement stage, calculated to be the sum of the van der Waals electrostatics and solvation energies, under the Generalized Born solvation model (GB/VI).

Table S9. Docking score and energy of the compounds and 1Hk7 Breast cancer protein.

Compound	S	rmsd_refine	E_conf	E_place	E_refine	E_score2
CTNZ	-6.66	1.07	123.12	-80.04	-15.59	-6.66
	-6.54	1.31	125.26	-54.53	-18.31	-6.54
	-5.69	1.04	162.33	-75.52	-6.83	-5.69
	-5.47	1.68	122.58	-57.66	-6.05	-5.47
	-5.44	2.13	123.29	-63.21	-6.84	-5.44
VO-CTNZ	-7.23	1.14	-756.05	-62.55	-40.37	-7.23
	-6.69	1.83	-694.59	-71.17	-15.75	-6.69
	-6.53	1.11	-712.89	-94.25	4.98	-6.53
	-6.52	1.39	-701.10	-75.22	-14.89	-6.52
	-6.40	1.21	-718.07	-63.05	-5.72	-6.40
MNZ	-8.09	1.59	23.59	-76.90	-44.16	-8.09
	-7.95	1.84	19.23	-70.98	-37.64	-7.95

	-7.88	1.57	26.80	-82.87	-40.81	-7.88
	-7.85	2.08	25.73	-82.01	-32.71	-7.85
	-7.78	2.47	22.75	-86.24	-33.18	-7.78
**VO-MNZ**	-11.09	1.36	-240.05	-32.65	-13.94	-11.09
	-10.57	2.21	-247.50	-37.12	-40.30	-10.57
	-10.25	4.65	-254.01	-72.24	-14.22	-10.25
	-10.19	2.83	-295.81	-44.12	-69.46	-10.19
	-10.13	2.52	-233.22	-36.05	-45.03	-10.13
**PNZ**	-8.59	2.04	36.47	-100.61	-45.99	-8.59
	-8.42	1.72	39.15	-109.48	-51.43	-8.42
	-8.35	2.28	42.02	-96.88	-45.93	-8.35
	-8.26	1.86	41.26	-96.16	-46.02	-8.26
	-8.25	1.16	39.35	-93.00	-38.43	-8.25
**VO-PNZ**	-11.30	1.51	-742.64	-109.33	7.32	-11.30
	-11.02	2.06	-720.81	-99.67	-7.31	-11.02
	-10.93	2.47	-731.11	-40.57	-21.73	-10.93
	-10.89	3.37	-693.13	-106.67	-42.32	-10.89
	-10.84	1.48	-735.85	-96.71	13.38	-10.84

Table S10. 2D and 3D Docking interaction of the VO(II) complexes with colon cancer protein "Human Thymidylate Synthase" for Hepatocellular carcinoma protein (PDB code = 1JU6) and Breast cancer (PDB code = 1HK7) proteins.

Types of Protein	Compound	2D snapshot	3D snapshot
**Hepatocellular carcinoma**	CTNZ	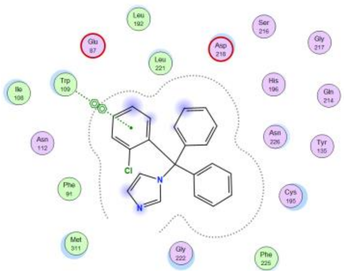	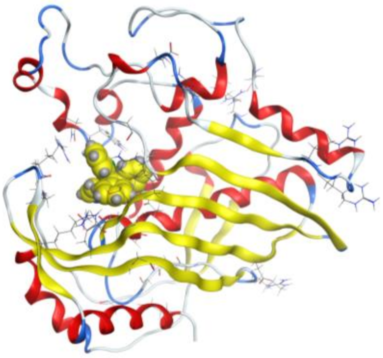
	VO-CTNZ	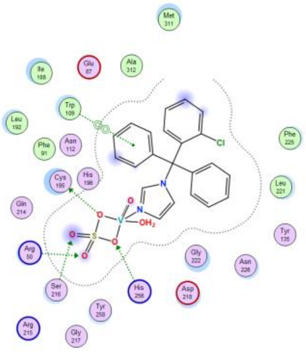	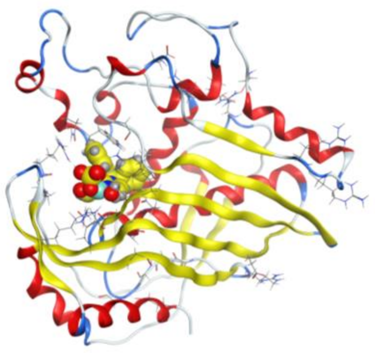

MNZ

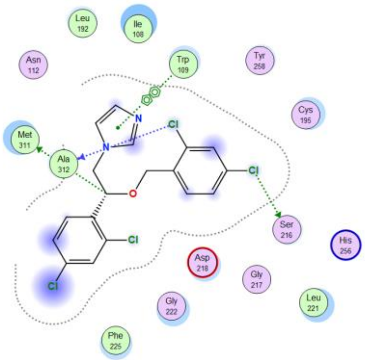

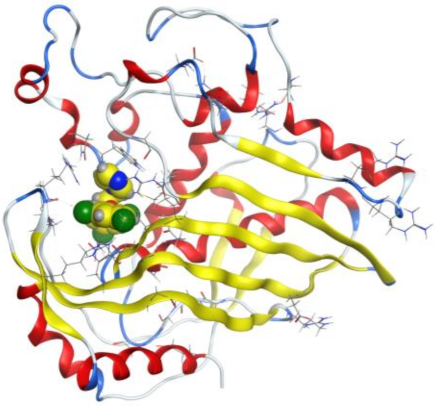

VO-MNZ

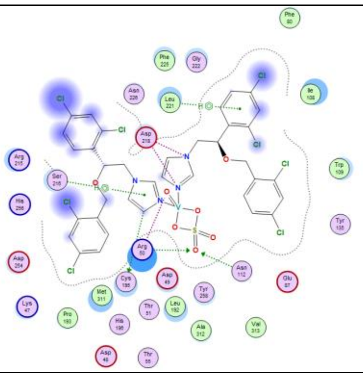

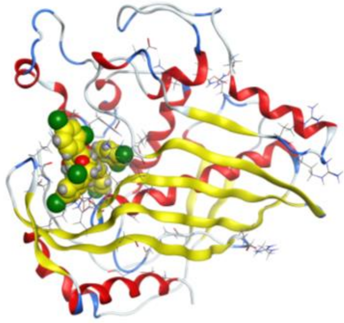

Breast cancer

CTNZ

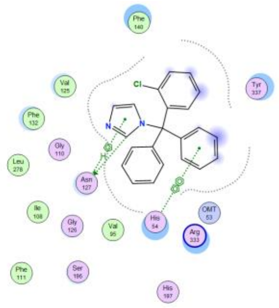

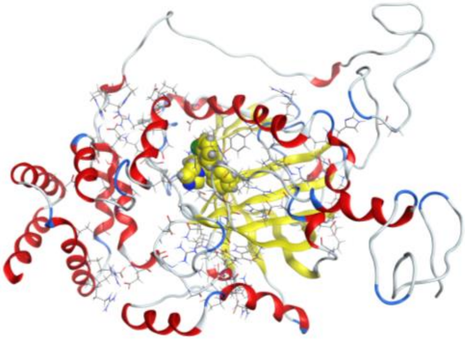

VO-CTNZ

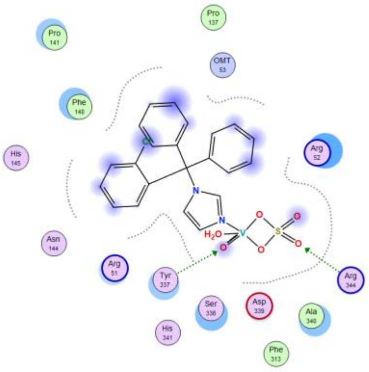

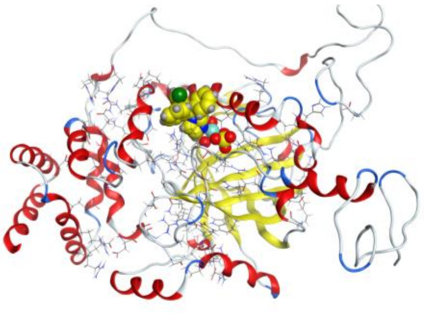

MNZ

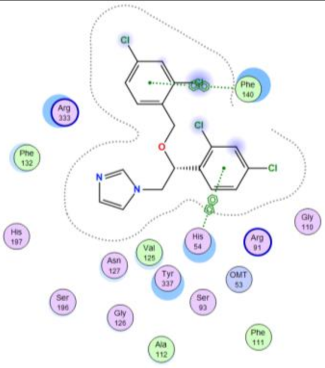

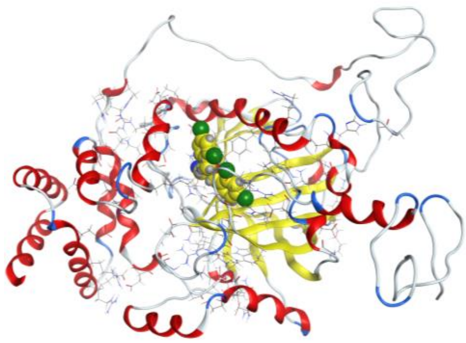

VO-MNZ	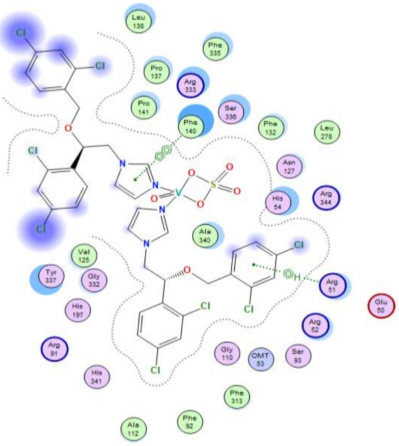	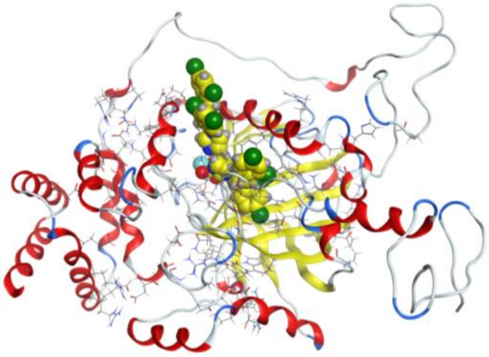

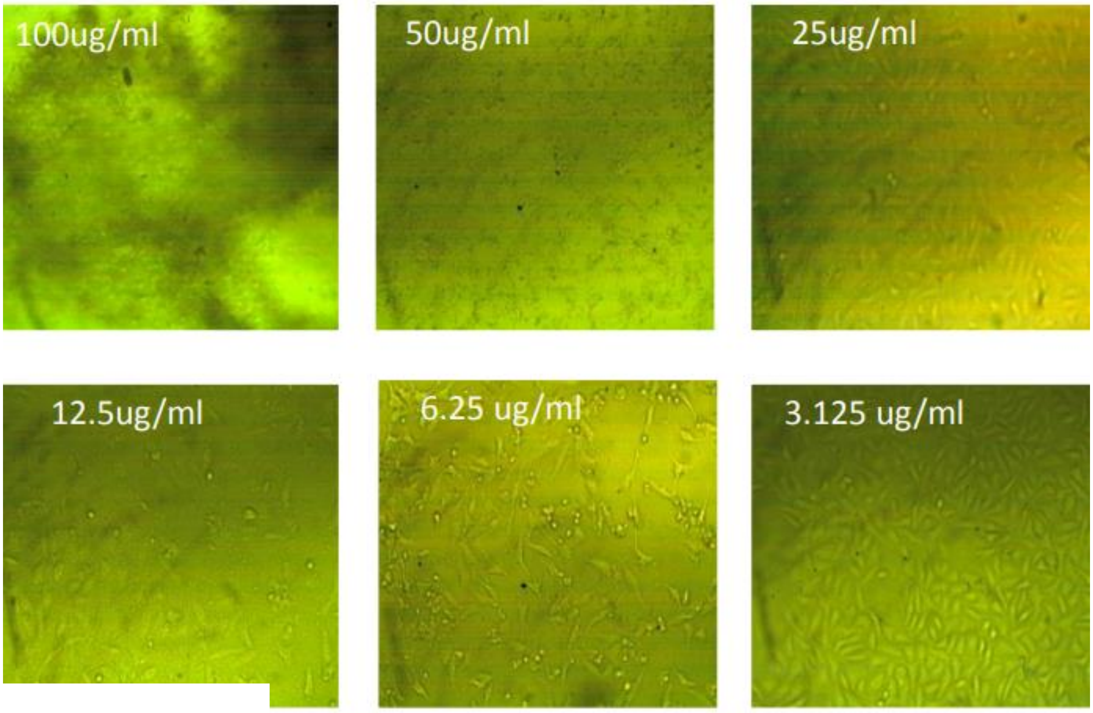

[VO(SO_4)(CTNZ)(H_2O)]H_2O

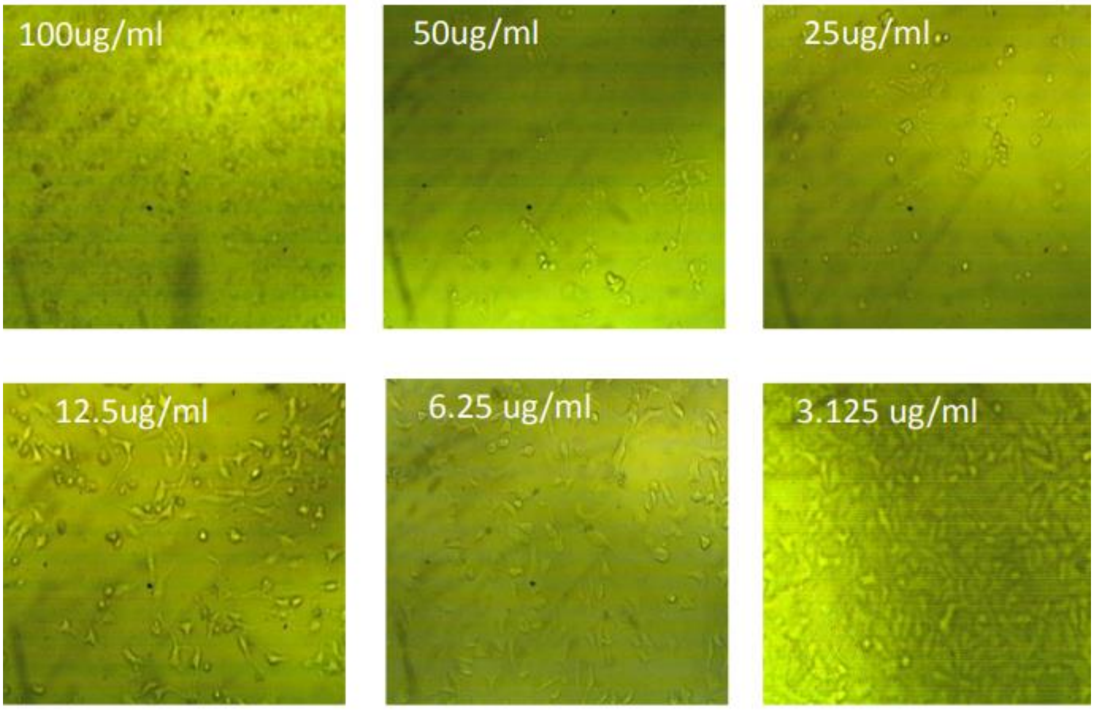

Figure S4. Morphology images of Hepatocellular carcinoma (HepG-2) treatment by $[VO(SO_4)(CTNZ)(H_2O)]H_2O$ and $[VO(SO_4)(MNZ)_2]$ H_2O.

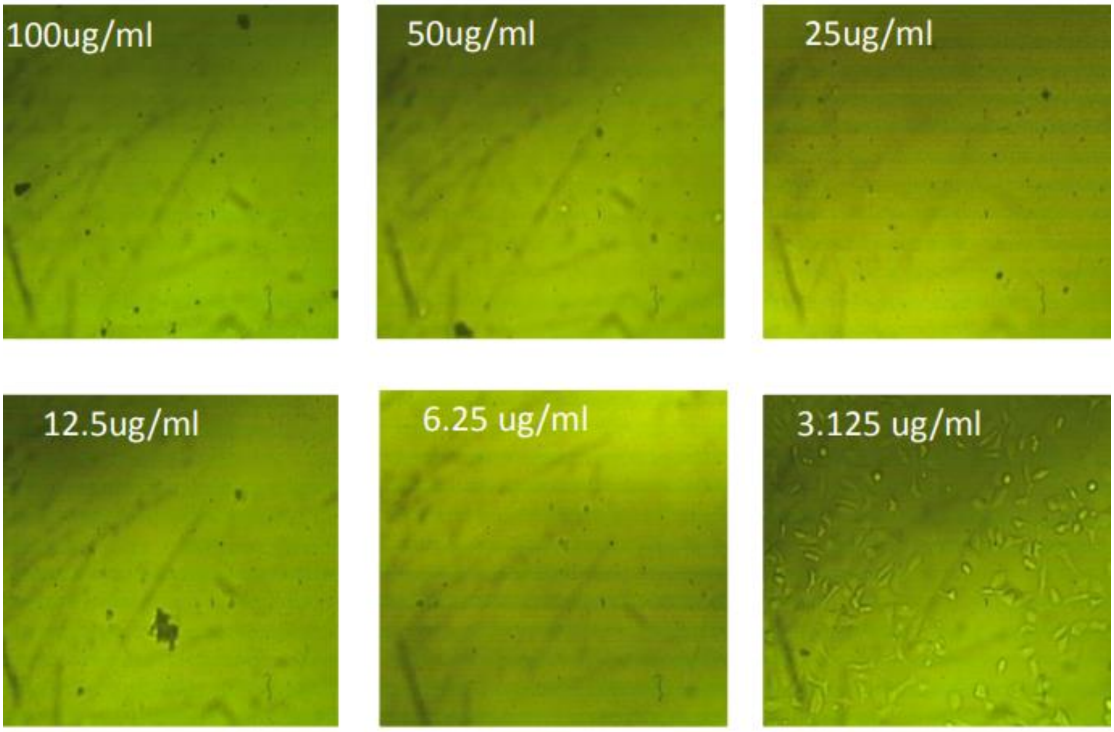

$[VO(SO_4)(CTNZ)(H_2O)]H_2O$

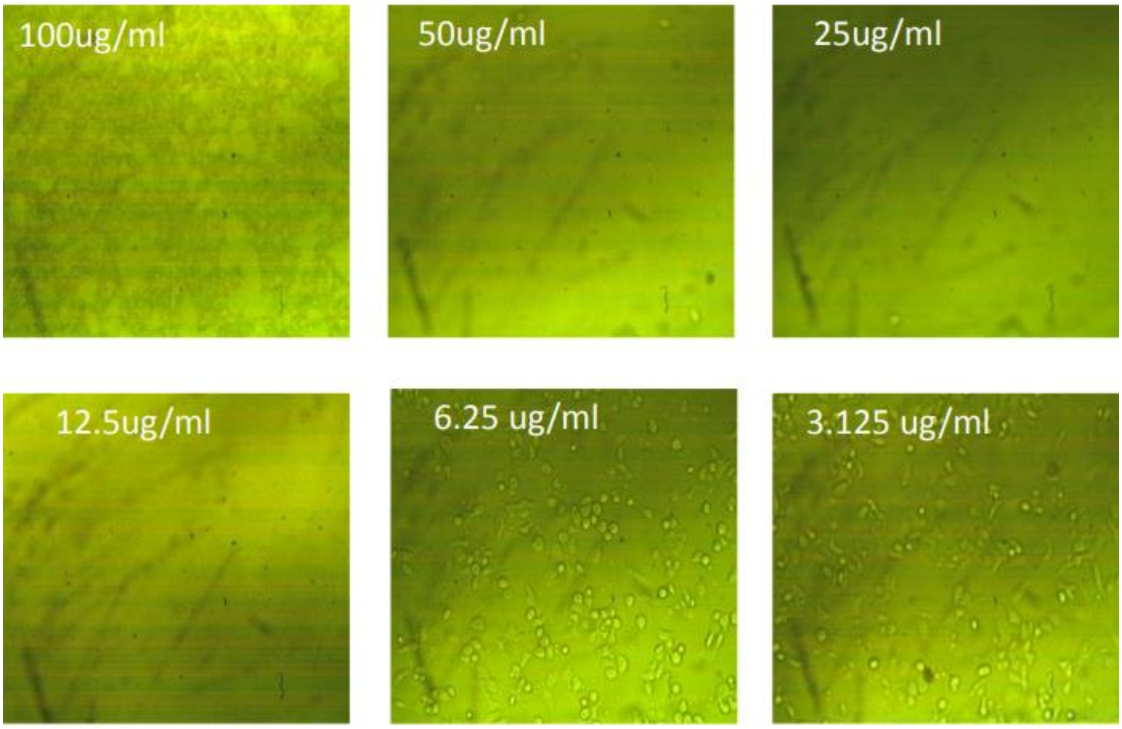

$[VO(SO_4)(MNZ)_2]$ H_2O

Figure S5. Morphology images of breast cancer (MCF-7) treatment by $[VO(SO_4)(CTNZ)(H_2O)]H_2O$ and $[VO(SO_4)(MNZ)_2]$ H_2O.
